# MAPK and GSK3/ß-TRCP-mediated degradation of the maternal Ets domain transcriptional repressor Yan/Tel controls the spatial expression of *nodal* in the sea urchin embryo

**DOI:** 10.1371/journal.pgen.1007621

**Published:** 2018-09-17

**Authors:** M. Dolores Molina, Magali Quirin, Emmanuel Haillot, Noémie De Crozé, Ryan Range, Mathieu Rouel, Felipe Jimenez, Radja Amrouche, Aline Chessel, Thierry Lepage

**Affiliations:** 1 Department of Natural Sciences, Institut Biologie Valrose, Université Côte d’Azur, Nice, France; 2 Department of Biological Sciences, Auburn University, Auburn, Alabama, United States of America; New York University, UNITED STATES

## Abstract

In the sea urchin embryo, specification of the dorsal-ventral axis critically relies on the spatially restricted expression of *nodal* in the presumptive ventral ectoderm. The ventral restriction of *nodal* expression requires the activity of the maternal TGF-β ligand Panda but the mechanism by which Panda restricts *nodal* expression is unknown. Similarly, what initiates expression of *nodal* in the ectoderm and what are the mechanisms that link patterning along the primary and secondary axes is not well understood. We report that in *Paracentrotus lividus*, the activity of the maternally expressed ETS-domain transcription factor Yan/Tel is essential for the spatial restriction of *nodal*. Inhibiting translation of maternal *yan/tel* mRNA disrupted dorsal-ventral patterning in all germ layers by causing a massive ectopic expression of *nodal* starting from cleavage stages, mimicking the phenotype caused by inactivation of the maternal Nodal antagonist Panda. We show that like in the fly or in vertebrates, the activity of sea urchin Yan/Tel is regulated by phosphorylation by MAP kinases. However, unlike in the fly or in vertebrates, phosphorylation by GSK3 plays a central role in the regulation Yan/Tel stability in the sea urchin. We show that GSK3 phosphorylates Yan/Tel in vitro at two different sites including a β-TRCP ubiquitin ligase degradation motif and a C-terminal Ser/Thr rich cluster and that phosphorylation of Yan/Tel by GSK3 triggers its degradation by a β-TRCP/proteasome pathway. Finally, we show that, Yan is epistatic to Panda and that the activity of Yan/Tel is required downstream of Panda to restrict *nodal* expression. Our results identify Yan/Tel as a central regulator of the spatial expression of *nodal* in *Paracentrotus lividus* and uncover a key interaction between the gene regulatory networks responsible for patterning the embryo along the dorsal-ventral and animal-vegetal axes.

## Author summary

Specification of the embryonic axes is an essential step during early development of metazoa. In the sea urchin embryo, specification of the dorsal-ventral axis critically relies on the spatial restriction of the expression of the TGF-ß family member Nodal in ventral cells, a process that requires the activity of the maternal determinant Panda. How the spatially restricted expression of *nodal* is established downstream of Panda is not well understood. We have discovered that, in the Mediterranean sea urchin *Paracentrotus lividus*, the spatial restriction of *nodal* on the ventral side of the embryo requires the inhibitory activity of a transcriptional repressor named Yan/Tel. This finding suggests a molecular mechanism for the control of *nodal* expression by the release of a repression. We found that this release requires the activity of two families of kinases that we identified as the MAP kinases and GSK3, a kinase which, intriguingly, was previously known as a key regulator of patterning along the animal-vegetal axis. We discovered that phosphorylation by MAPK and GSK3 triggers degradation of Yan/Tel by a β-TRCP proteasome pathway. Finally, we find that Yan/Tel likely acts downstream of Panda in the hierarchy of genes required for *nodal* restriction. Our study therefore identifies Yan/Tel as a new essential regulator of *nodal* expression downstream of Panda and identifies a novel key interaction between the gene regulatory networks responsible for patterning along the primary and secondary axis of polarity.

## Introduction

In the sea urchin embryo, specification of the dorsal-ventral (D/V) axis critically relies on zygotic expression of the gene encoding the TGF-β family member Nodal in the presumptive ventral ectoderm [1]. Nodal signaling promotes specification of the ventral ectoderm and triggers expression of BMP2/4, which acts as a long-range morphogen that specifies and patterns the dorsal region [2]. Sea urchin embryos lacking Nodal function develop with a strongly radialized phenotype and fail to express both ventral and dorsal marker genes. However, injection of *nodal* mRNA into a single cell can rescue a complete dorsal-ventral axis in these *nodal* morpholino injected embryos [1]. Therefore, expression of *nodal* is a key event that launches the gene regulatory network that specifies the D/V axis and understanding how *nodal* expression is regulated is essential to understand how secondary axis specification is established [3].

*nodal* expression is initiated around the 32/64 cell-stage and is initially rather broad, encompassing most cells of the presumptive ectoderm. Starting at the early blastula stage, *nodal* expression is progressively restricted to a smaller domain that corresponds to the presumptive ventral ectoderm [4]. The progressive restriction of *nodal* expression in the sea urchin embryo is thought to rely on the ability of Nodal to stimulate its own expression as well as that of the *lefty* gene, which encodes a diffusible long-range inhibitor of Nodal signaling [5–8]. Together, the auto-activation of Nodal and the long-range inhibition of Lefty constitute the basis for a reaction-diffusion mechanism, i.e. a system with self-organizing properties, able to amplify weak initial anisotropies and to generate sharp boundaries within a field of cells [9–14]. In the absence of Lefty, *nodal* expression is not restricted ventrally and expands towards the dorsal ectoderm [14]. Although this reaction-diffusion mechanism is clearly essential for the spatial restriction of *nodal*, Lefty is not the first factor acting in the process leading to the progressive restriction of *nodal* expression. The spatial restriction of *nodal* was recently shown to critically rely on the activity of a maternal TGF-ß ligand named Panda [15]. When maternal but not zygotic *panda* function is blocked, *nodal* is ectopically expressed from the very beginning of its expression until late in gastrulation despite the presence of Lefty, indicating that the reaction-diffusion mechanism between Nodal and Lefty is not sufficient to break the initial radial symmetry of the embryo. However, *panda* morphants nevertheless recover a dorsal-ventral axis late in gastrulation due to compensatory zygotic mechanisms. Maternal *panda* transcripts appear to be asymmetrically distributed in the egg and indeed, local overexpression of *panda* into one blastomere, but not global overexpression of *panda* into the egg, rescues the D/V polarity of *panda* morphants. The mechanism by which Panda restricts *nodal* expression is not elucidated but an intriguing hypothesis is that Panda may act by down-regulating the Nodal amplification loop on the dorsal side [15]. In addition to Panda and Lefty, the activity of the maternal Hypoxia induced transcription factor HIF-1 alpha has been proposed to contribute to the spatial restriction of *nodal* in *S*. *purpuratus*. However, HIF-1 alpha does not appear to be a crucial factor to establish the spatial expression of *nodal* and embryos lacking HIF-1 alpha only show a transient increase in *nodal* expression at early blastula stage but express *nodal* in a spatially restricted manner thereafter and develop with a normal dorsal-ventral axis [16].

Intriguingly, the absence of Lefty or of Panda does not cause ectopic expression of *nodal* in other territories that normally do not express *nodal* such as the animal pole region and the endomesoderm indicating that, in addition to Lefty and Panda, other factors likely contribute to the repression of *nodal* expression in these domains. The transcription factor FoxQ2 has been identified recently as one such factor that acts redundantly with Lefty to prevent *nodal* expression in the animal pole [17, 18]. *foxQ2* transcripts are expressed in the animal half of the embryo during cleavage but are progressively restricted to the animal pole region by a Wnt/β-Catenin-dependent process [18, 19]. Interfering with *foxQ2* mRNA translation does not perturb *nodal* expression or D/V axis formation, but disrupts specification of animal pole region and the resulting embryos lack the animal most territory called the apical tuft. In the double *lefty*+*foxQ2* morphants, *nodal* expression expands to the animal pole region suggesting that Lefty and FoxQ2 act redundantly to repress *nodal* expression in the animal pole region. Conversely, *nodal* is not expressed in embryos with an expanded animal pole that retain *foxQ2* expression such as in embryos animalized by inhibition of vegetal signaling. However, preventing translation of the *foxQ2* transcript in these embryos is sufficient to rescue *nodal* expression and to restore normal patterning of the ectoderm. On the basis of these observations, it was suggested that in the unperturbed embryo, FoxQ2 links D/V axis formation to animal-vegetal patterning through its repressive action on *nodal* or in other words, that the vegetal signaling initiated by canonical Wnt/β-Catenin plays a permissive role in D/V axis formation by releasing the repressive action of FoxQ2 on *nodal* [17, 18]. However, as mentioned above, inhibition of *foxQ2* mRNA translation has no effect on the timing or the spatial regulation of *nodal* expression suggesting that FoxQ2 is not essential for the timing or the spatial expression of *nodal* in the unperturbed embryo. Consistent with this idea, FoxQ2 protein is not detectable in the early embryo and only appears in the nuclei of animal pole cells of embryos at late blastula stage, i.e. after the establishment of the spatial expression of *nodal*. Therefore, how the spatial expression of *nodal* is initiated and how *nodal* expression along the D/V axis is coordinated with patterning along the animal-vegetal axis remains not well understood.

In this study, we report that the ETS domain transcriptional repressor Yan/Tel is an essential maternal regulator of *nodal* expression that links animal-vegetal signaling to the spatial expression of *nodal* during dorsal-ventral axis formation in *Paracentrotus lividus*. Furthermore, we identify the kinase GSK3 as a key component of the animal-vegetal patterning system that regulates the stability of Yan/Tel by triggering its degradation by a β-TRCP proteasome pathway. Finally, we show that Yan/Tel is epistatic to Panda and that Panda overexpression affects the phosphorylation state of Yan/Tel strongly suggesting that Panda contributes to the stabilization/regulation of Yan/Tel to regulate *nodal* expression. These results identify Yan/Tel as a new central regulator of *nodal* acting downstream of Panda and show that degradation of Yan/Tel by GSK3-mediated phosphorylation in the animal hemisphere is the key event that allows *nodal* expression and links patterning along the primary and secondary axes.

## Results

### ETS transcription factors repress the activity of the *nodal* promoter

**Fig 1:**
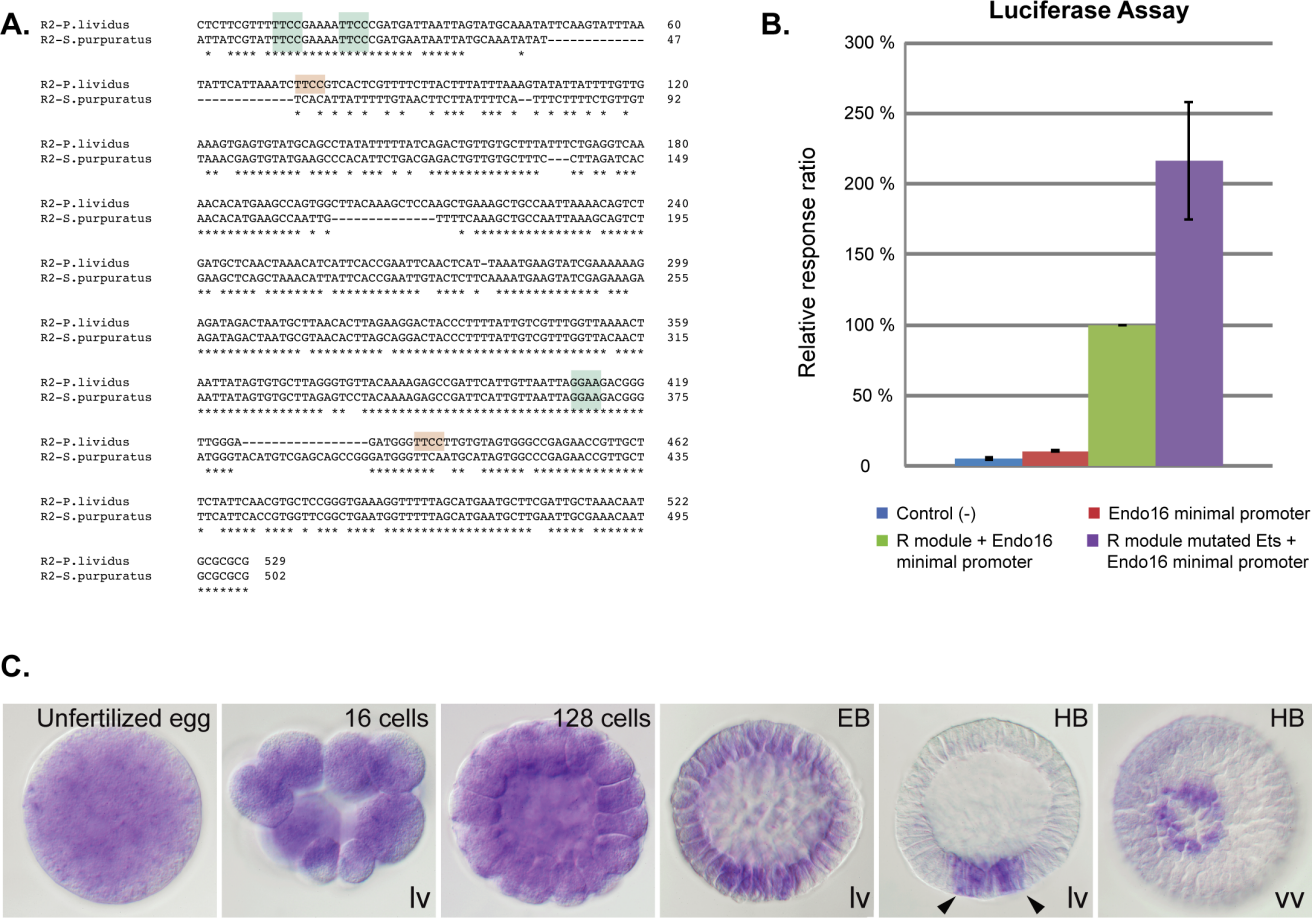
Repression of the proximal promoter region of *nodal* by ETS factors and expression pattern of Yan/Tel. A, Alignment of the proximal cis-regulatory module of *nodal* (R module) of the sea urchin species *Paracentrotus lividus* and *Strongylocentrotus purpuratus* showing the conservation of ETS binding sites (highlighted in light green). Two extra ETS binding sites are present in the cis-regulatory module of *P*. *lividus* (highlighted in light brown). B, Luciferase assays showing that mutations of putative ETS sites increase the transcriptional activity of the *nodal* promoter. The data are presented as the ratio of luciferase expression between each construct and the wild-type R module (green). The bars on the graphs represent the standard error. C, Expression pattern of *yan/tel* during normal development. After a phase of maternal ubiquitous expression, *yan/tel* is strongly expressed in the precursors of the skeletogenic mesoderm (arrowheads at HB stage). EB, early blastula; HB, hatching blastula; vv, vegetal view; lv, lateral view. In the lateral view, animal is to the top.

In the course of a cis-regulatory analysis of the *nodal* promoter, we noticed that, in addition to bZIP, Sox, Oct, homeobox and Smad binding sites, the 5' proximal module of the *nodal* promoter, called the R2 module, contains several conserved 5'-GGAA/T-3' ETS binding motifs, the function of which had not been analyzed previously (Fig 1A) [4]. To test the role of these sites in the transcriptional activation of *nodal*, we injected luciferase reporter constructs into fertilized sea urchin eggs and compared the activity of the wild-type construct with that of a mutant reporter lacking these ETS sites at blastula stage. Surprisingly, mutation of the 5 ETS sites contained in the 5' module did not reduce but instead significantly increased the activity of the *nodal* 5' proximal module, which reached up to 2 fold its normal value (Fig 1B). This suggested that members of the ETS family with transcriptional repressor activity negatively control *nodal* expression by binding to the proximal module of its promoter.

### Sea urchin Yan/Tel is ubiquitously expressed maternally while early zygotic expression is restricted to the mesoderm

The sea urchin genome contains eleven genes encoding ETS transcription factors and some of them are presumed to be transcriptional repressors [20, 21]. We focused on the gene encoding the ETS transcription factor Yan/Tel since this gene is known to encode a prototypical repressor [22–26] (reviewed in [27]).

The overall structure of sea urchin Yan/Tel protein is conserved with an N-terminal SAM domain, a central co-repressor binding domain and a C-terminal ETS DNA binding domain (S1A Fig). The central domain is thought to be required for the recruitment of the co-repressors mSin3A, SMRT and N-CoR, which in turn can recruit histone deacetylases [28–30]. In addition, the N-terminal SAM domain of Yan and Tel oligomerizes in a head to tail manner and this polymerization has been shown to be essential for repression [31–33]. Interestingly, most of the hydrophobic residues that make up the interfaces between two monomers of Yan from *Drosophila* or Tel from vertebrates are conserved in the sea urchin Yan/Tel protein (S1B Fig), suggesting that sea urchin Yan/Tel may also form higher order polymers as do its vertebrate and fly orthologues.

Tel has been shown to be sumoylated by UBC9 on Lysine 11 and 99 and this modification has been shown to downregulate Tel and to promote its nuclear export. One of these residues, lysine 99, is conserved in the sea urchin Yan/Tel suggesting that it may also be regulated by sumoylation (S1 Fig). Yan and Tel have also been shown to be degraded primarily following ubiquitination by the ubiquitin conjugating enzyme FBL6. However, the Ubiquitin acceptor sites of Tel have not been identified with certainty. Finally, both Yan and Tel have been shown to be phosphorylated by MAP kinases at multiple residues and this phosphorylation has been shown to downregulate Yan and Tel by promoting degradation and/or reducing DNA binding [22–26]. Intriguingly, sea urchin Yan/Tel also contains three canonical PXS/TP phosphorylation sites for MAP kinases but the positions of these sites do not appear to be conserved between the sea urchin, vertebrate and *Drosophila* proteins (S1C Fig).

Northern blot analysis and in situ hybridization revealed that *yan/tel* is a maternal transcript expressed abundantly and ubiquitously during cleavage (Fig 1C and S2 Fig). Starting late in cleavage and during prehatching and hatching blastula stages, zygotic *yan/tel* transcripts were detected in a small ring of cells located in the vegetal pole region that likely corresponds to precursors of the skeletogenic mesoderm (arrowheads in Fig 1C).

### The transcriptional repressor Yan/Tel is essential for dorsal-ventral axis formation

**Fig 2:**
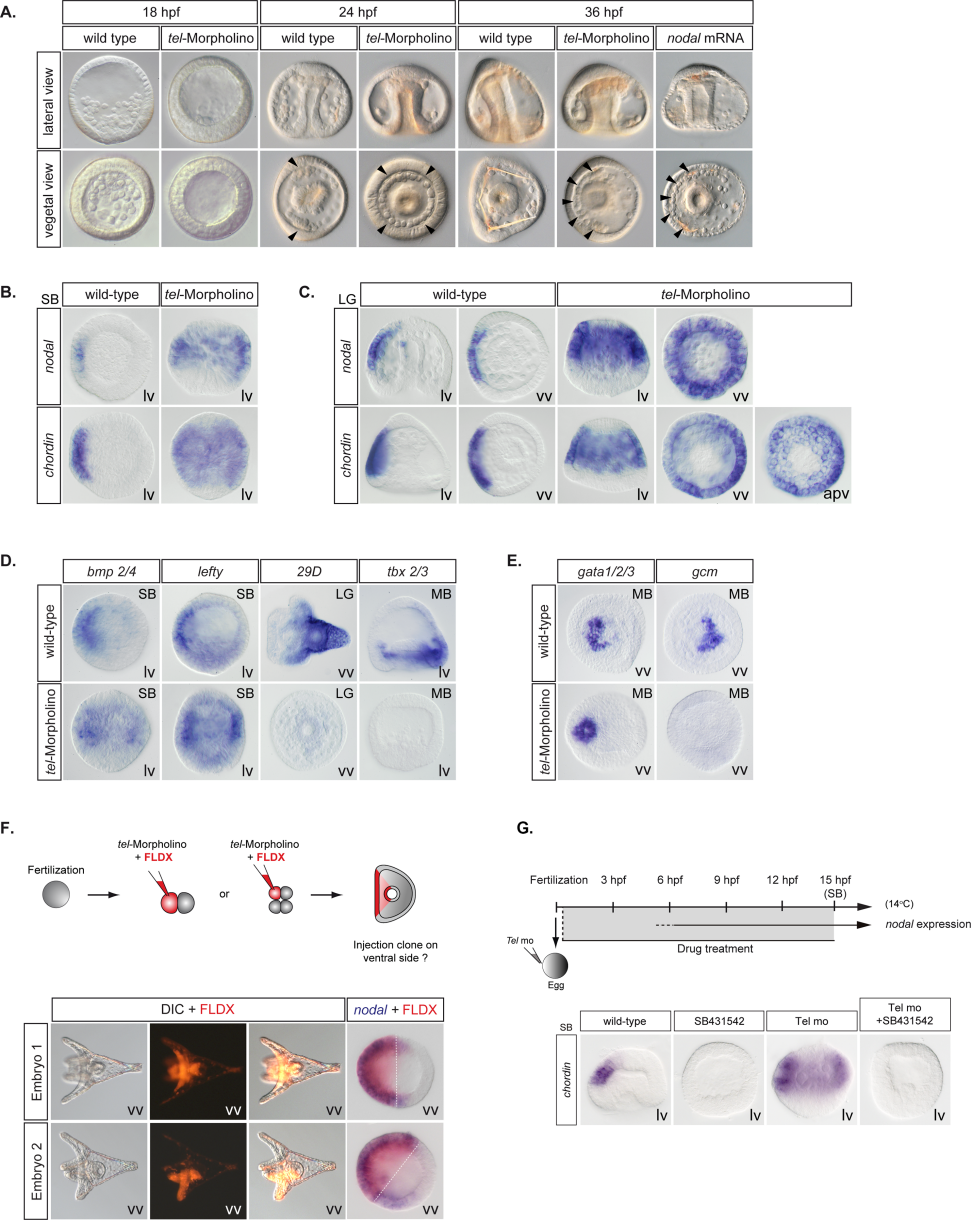
Yan/Tel controls the spatial restriction of *nodal* expression in the ectoderm. A, Inhibition of maternal Yan/Tel function disrupts dorsal-ventral axis formation. Note the radial arrangement of the primary mesenchymal cells (PMCs, arrowheads) at gastrula stage (24hpf) and the rounded shape of the embryo at prism stage (36hpf). (hpf), hours post-fertilization. B-C, Inhibition of *yan/tel* mRNA translation causes a massive ectopic expression of *nodal* and *chordin* at blastula (B) and gastrula (C) stages. Note that the expression has expanded throughout most of the ectoderm but is excluded from the animal pole region. apv, animal pole view. D, Inhibition of *yan/tel* mRNA translation expands the expression of the ventral ectodermal markers *bmp2/4* and *lefty* and suppresses the expression of the dorsal ectodermal markers *29D* and *tbx2/3*. E, Inhibition of *yan/tel* mRNA translation expands the expression of the ventral mesodermal marker *gata1/2/3* and suppresses the expression of dorsal mesodermal marker *gcm*. F, Random local inhibition of Yan/Tel function orients the dorsal-ventral axis. In all embryos injected randomly with the *yan/tel* morpholino into one blastomere at the 2 or 4-cell stage, *nodal* expression (blue) overlaps with the progeny of the injected blastomere (red). G, Ectopic expression of the Nodal downstream target gene *chordin* following inhibition of *yan/tel* mRNA translation depends on Nodal pathway activity. Note that inhibition of *yan/tel* function followed by treatment with the Nodal receptor inhibitor SB431542 blocks the ectopic expression of *chordin* observed in y*an/tel* morphants. SB, swimming blastula stage; LG, late gastrula stage. MB, mesenchyme blastula stage; vv, vegetal view; lv, lateral view. In the lateral views, animal is to the top, and ventral to the left.

To determine if Yan/Tel is the factor responsible for repressing the activity of the *nodal* promoter, we tested the effects on *nodal* expression of injecting antisense morpholino oligonucleotides against *yan/tel*. Embryos injected with morpholinos directed against the translation start site of the *yan/tel* transcript developed normally up to the late blastula stage. Then, while in control embryos precursors of the primary mesenchymal cells (PMCs) ingressed into the blastocoel, in *yan/tel* morphants, ingression of the PMCs was clearly delayed. At the early gastrula stage (24hpf), while in the control embryos D/V polarity was already apparent by the presence of bilateral clusters of PMCs (arrowheads) containing spicule rudiments and by the flattening of the presumptive ventral side, in the *yan/tel* morphants, no visible sign of dorsal-ventral polarity was apparent: the PMCs remained arranged in a ring around the archenteron (arrowheads in Fig 2A) and the embryos conserved a rounded shape. This apparent lack of dorsal-ventral polarity persisted at the late gastrula stage: when in control embryos the archenteron bent toward the presumptive ventral ectoderm, the *yan/tel* morphants remained radialized as evidenced by the straight position of the archenteron at the center of the blastocoel and by the radial arrangement of the PMCs. Surprisingly, despite this apparent complete failure to establish a D/V axis until late in gastrulation, the *yan/tel* morphants progressively recovered a D/V polarity and at the equivalent of the early prism stage (36 hpf), the first signs of dorsal-ventral polarity appeared in these embryos. A thickened ventral-like ectoderm started to differentiate on one half of the embryo and the archenteron adopted a slightly asymmetrical position closer to this thickened ectoderm. However, instead of forming bilateral clusters, the PMCs arranged themselves in an extended half-circle at the basis of the ventral ectoderm. Interestingly, the morphology of *yan/tel* morphants at this stage was similar to that of embryos partially ventralized by treatment with recombinant Nodal or by overexpression of low doses of *nodal* mRNA or to that of embryos lacking the function of the maternal factor Panda that is required to restrict *nodal* expression to the ventral side (Fig 2A)[15]. These strong effects on D/V axis formation were observed following injection of two different morpholino oligonucleotides targeting either the translation start site or the 5’ UTR and were completely suppressed by co-injection into the egg of a wild-type form of *yan/tel* mRNA immune against the morpholino (S3 Fig). In contrast, injection of a morpholino oligonucleotide targeting a splice junction abrogated skeletogenesis (S4 Fig), consistent with the zygotic expression of the gene in the PMCs, but did not affect establishment of the dorsal-ventral axis and did not prevent formation of the bilateral PMC clusters suggesting that the maternal function of Yan/Tel but not its zygotic function is required for establishment of the dorsal-ventral axis.

To understand the origin of the radialized phenotype of *yan/tel* morphants, we examined the expression of *nodal*, *chordin* and *bmp2/4* as well as of some of their downstream targets in the ectoderm such as *29D* and *tbx2/3* (Fig 2B–2D). Strikingly, consistent with their radialized phenotype and with their resemblance to *panda* morphants, *yan/tel* morphants displayed a dramatic ectopic expression of *nodal* as well as of *chordin*, *bmp2/4* and *lefty*, three direct downstream targets of Nodal signaling [34]. Interestingly, this ectopic expression was observed within a large ectodermal domain that encompassed the presumptive ciliary band and dorsal ectoderm, forming a large belt of cells around the embryo, but it did not extend to the animal pole region or to the endomesoderm territory (Fig 2C). Also, consistent with the observed expansion of *nodal* expression, the expression of dorsal markers such as *tbx2/3* and *29D* was either eliminated or dramatically reduced in the *yan/tel* morphants (Fig 2D). Dorsal-ventral patterning of the mesoderm was also strongly perturbed in the *yan/tel* morphants, which typically contained very few pigment cells. Indeed, the ventral expression of the blastocoelar cell marker *gata1/2/3* was radialized while expression of the dorsally expressed pigment cell marker *gcm* was suppressed (Fig 2E), in agreement with the radialized expression of *nodal* resulting from inhibition of *yan/tel* mRNA translation [35].

Since *yan/tel* morphants ectopically express *nodal*, we tested if local inhibition of *yan/tel* mRNA translation is sufficient to trigger *nodal* expression and to orient the dorsal-ventral axis. Since in *Paracentrotus* the orientation of the D/V axis is not related to the plane of first cleavage, we injected the *yan/tel* morpholino randomly into one blastomere of embryos at the 2 or 4 cell-stage and later scored the position of the clone of injected cells at pluteus stage. In most of the injected embryos (n>50), *nodal* expression was later found in a territory either congruent or overlapping with the clone of cells derived from the injected blastomere (Fig 2F). Remarkably, the boundaries of the clone were always included in the *nodal* expressing territory and at least one of these boundaries always precisely aligned with the border of the *nodal* expression domain. Therefore, blocking translation of Yan/Tel mRNA caused the cell-autonomous expression of *nodal*, irrespective of the position of the clone. At pluteus stage, the progeny of the injected cells was always found on the ventral side of the larva. These results demonstrate that Yan/Tel is an essential regulator of *nodal* in vivo. They also strongly suggest a model of *nodal* activation by release of Yan/Tel-mediated transcriptional repression.

Finally, since *yan/tel* morphants displayed a dramatic ectopic expression of *nodal* as well as of *chordin*, *bmp2/4* and *lefty*, we tested if Yan/Tel exerts a global repression on several genes of the Nodal pathway. Treatment with the Nodal receptor inhibitor SB431542 blocked the ectopic expression of *chordin* observed in *yan/tel* morphants (Fig 2G). Therefore, the ectopic expression of the Nodal downstream target genes like *chordin* following inhibition of *yan/tel* is most likely indirect and depends on Nodal pathway activity. *nodal* is likely the main gene of the Nodal pathway whose expression is directly regulated by Yan/Tel.

Taken together these results show that in addition to the reaction-diffusion mechanism based on Lefty and to the activity of the maternal TGF-ß ligand Panda recently shown to be essential for the spatial restriction of *nodal*, the transcriptional repressor Yan/Tel plays a central role in dorsal-ventral patterning of the ectoderm and mesoderm by regulating the spatial expression of *nodal*.

### Yan/Tel acts upstream of the Lefty-dependent reaction-diffusion mechanism to initiate the spatial restriction of *nodal*

**Fig 3:**
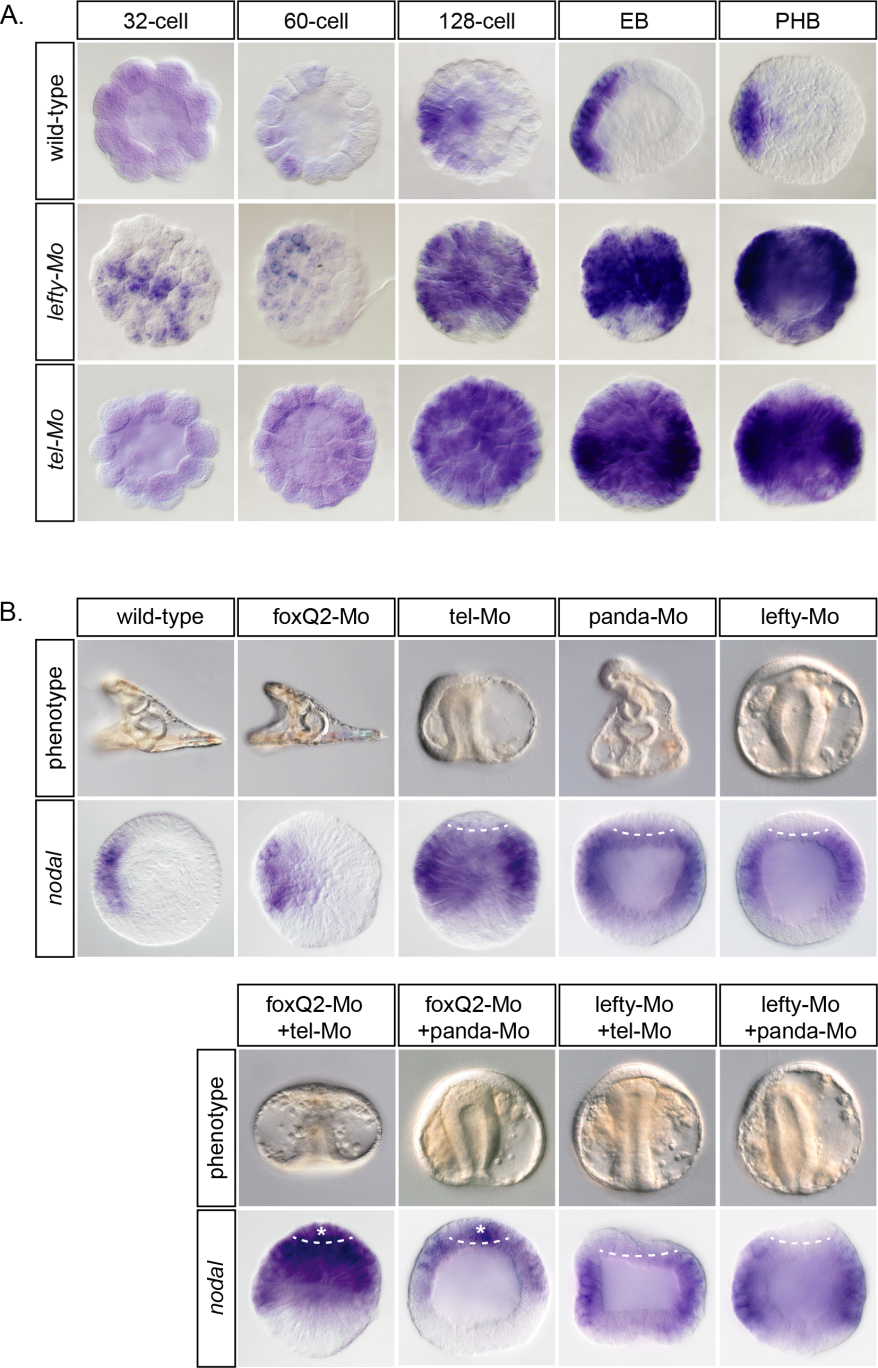
Yan/Tel is required before Lefty to restrict *nodal* expression and acts redundantly with FoxQ2 in the animal pole region. A, Time-course of *nodal* expression in control, *lefty* morpholino and *yan/tel* morpholino injected embryos. Note that ectopic expression of *nodal* is detected in the *yan/tel* morphants earlier than in the *lefty* morphants. (EB), early blastula; (PHB), pre-hatching blastula. B, Note that *nodal* is ectopically expressed in the animal pole region (asterisks) of embryos co-injected with *foxQ2* and *tel* or *panda* morpholinos, but absent from this region after co-injection of the *lefty* and *panda* or *yan/tel* morpholinos. All views are lateral. Animal is to the top, and ventral to the left.

Previous studies showed that the spatial restriction of *nodal* expression relies on the early establishment of a reaction-diffusion mechanism between Nodal and Lefty starting at the early blastula stage [1, 14, 36]. To determine if the function of Yan/Tel is required early during cleavage stages for the establishment of the spatial restriction of *nodal* or if it is only necessary at later stages for its maintenance, we performed a time-course experiment and compared *nodal* expression in *lefty* morphants and *yan/tel* morphants. Embryos were injected with either the *lefty* morpholino or the *yan/tel* morpholino and the expression of *nodal* was examined at successive stages starting at 32-cell stage, when *nodal* expression is first detectable in a broad domain, up to pre-hatching blastula, when its expression is sharply restricted to the presumptive ventral ectoderm. About half of the *lefty* morphants embryos at 60-cell stage displayed a localized *nodal* expression indicating that the spatial restriction of *nodal* was initiated normally in these embryos (Fig 3A). Nevertheless, in the absence of Lefty, the spatial restriction of *nodal* expression was not maintained at later stages and most embryos eventually showed a massive ectopic expression of *nodal*, as reported previously [14]. In contrast, in the *yan/tel* morpholino injected embryos, no restricted expression of *nodal* was visible at any stage and all the injected embryos showed a massive ectopic expression of *nodal* from early on (Fig 3A). These results suggest that the function of Yan/Tel is required before that of Lefty for the establishment of the spatial restriction of *nodal*. Although its expression is not restricted along the D/V axis, Yan/Tel is required for the initial restriction of *nodal* expression while the function of Lefty appears to be required only later for the maintenance of the spatial restriction of this gene.

### Yan/Tel cooperates with FoxQ2 to repress *nodal* expression in the animal pole domain

Both in the unperturbed embryo and following overexpression of *nodal*, *nodal* expression is not detected in the animal pole [1, 14, 15, 18, 34, 36–38]. The animal pole therefore appears as a territory refractory to *nodal* expression. It was shown previously that *foxQ2*, which is expressed specifically in the animal pole, acts together with Lefty to repress *nodal* expression in this region [18]. Since Yan/Tel is a repressor of *nodal* expression and since it is expressed ubiquitously at early stages, we tested if its function is required to repress *nodal* expression in the animal pole domain. As shown previously [1, 15, 18], embryos injected with morpholinos directed against *foxQ2* or *panda* or *lefty* transcripts did not show ectopic expression of *nodal* in the animal pole domain (Fig 3B). In contrast, in embryos co-injected with either the *foxQ2* and *yan/tel* or with *foxQ2* and *panda* morpholinos, *nodal* expression expanded dramatically to fill up the animal pole domain (asterisks in Fig 3B). This suggests that in the *foxQ2* morphants, *nodal* expression does not expand to the animal pole region because the activities of Yan/Tel and Panda prevent expansion of *nodal* in this region. It was shown previously that co-injection of *foxQ2* and *lefty* morpholinos resulted in ectopic expression of *nodal* in the animal pole domain [18]. In contrast, embryos co-injected with *yan/tel* and *lefty* morpholinos or with *panda* and *lefty* morpholinos did not show an expansion of *nodal* expression in the animal pole region (Fig 3B). This suggests that although Yan/Tel, Lefty and Panda cooperate with FoxQ2 to repress *nodal* expression in the animal pole, the contribution of these factors in the repression of *nodal* may not be as crucial as that exerted by FoxQ2 since only on the absence of FoxQ2 is *nodal* ectopically expressed in the animal pole region.

Taken together these results show that Yan/Tel plays an important role as a repressor of *nodal* expression in the animal pole domain where it acts together with FoxQ2, Lefty and Panda. While removing the function of either gene alone is not sufficient to cause ectopic expression of *nodal* in the animal pole region, removing the function of Yan/Tel and FoxQ2 triggers ectopic expression of *nodal* in this region.

### Multiple phosphorylation sites regulate the stability of sea urchin Yan/Tel

**Fig 4:**
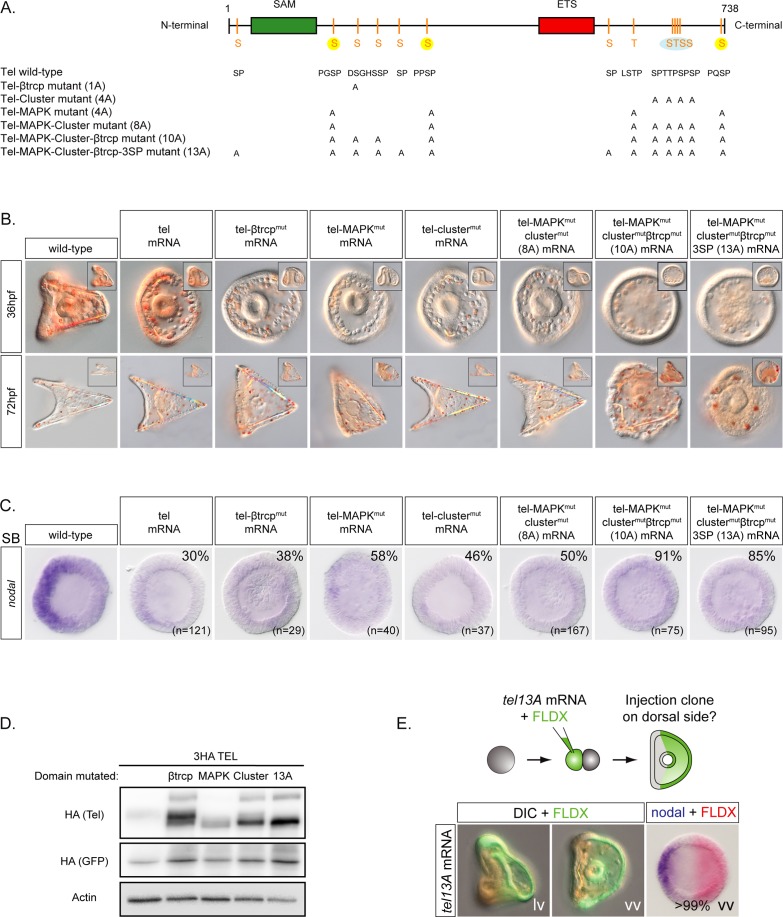
Phosphorylation of Yan/Tel by MAPKs regulates *nodal* expression. A, Structure of wild-type Yan/Tel and of phosphorylation site mutant forms of Yan/Tel used in this study. The Yan/Tel protein contains a SAM domain, an ETS binding site domain and several MAPK consensus phosphorylation sites indicated as S or T. The 3 canonical consensus MAPK phosphorylation sites are highlighted in yellow and the cluster of 4 putative phosphorylation sites is highlighted in blue. B, Overexpression of a wild-type form of Yan/Tel causes little effects on development of the embryos, while overexpression of phosphorylation mutant forms of Yan/Tel progressively radializes the embryos. The phosphorylation mutant *yan/tel10A* produces a phenotype similar to the *nodal* loss-of-function phenotype: note the radial arrangement of the PMCs, the abundance of pigment cells, the straight archenteron and the thickened ectoderm. Overexpression of the phosphorylation mutant form *yan/tel13A* fully radializes the embryo and disrupts gastrulation. All images are vegetal views at pluteus stage. Lateral views of the same embryo are shown in the upper corner. C, Overexpression of the phosphorylation mutant forms of *yan/tel* increase the percentage of embryos that fail to express *nodal* compared to overexpression of wild-type *yan/tel* mRNA. Embryos are shown at swimming blastula stage (SB) and are vegetal views. D, Western blot against HA-tagged versions of wild-type and mutant Yan/Tel. Mutation of the β-TRCP degradation motif does not change significantly the migration of Yan/Tel but results in a marked stabilization of the protein. In contrast, MAPK, cluster and yan/tel13A phosphorylation mutants migrate predominantly as a fast migrating isoform. HA-Tagged GFP mRNA was co-injected as a control. E, Random injection of a phosphorylation mutant form of Yan/Tel efficiently orients the dorsal-ventral axis. DIC and fluorescent images of an embryo injected randomly into one blastomere at the 2-cell stage with the *yan/tel13A* mRNA. In all embryos injected, *nodal* expression (blue) at swimming blastula stage is systematically found on the side opposite to the injection clone (red). vv, vegetal view; lv, lateral view, with animal to the top, and ventral to the left.

Overexpression of wild-type *yan/tel* mRNA had only moderate effects on development of sea urchin embryos (Fig 4B). Even when injected with high doses (1000 μg/ml) of this mRNA, the embryos developed into pluteus larvae and only occasionally, reduced D/V axis and ectopic skeletal elements were observed. Following overexpression of wt *yan/tel* mRNA, *nodal* expression was absent or strongly reduced during blastula stages in 30% of the embryos but it was progressively restored to normal levels during gastrula stages consistent with the apparent normal morphology of *yan/tel* overexpressing embryos at pluteus stages (Fig 4B and 4C). This result is consistent with previous studies showing that the activity of *Drosophila* Yan or of vertebrate Tel is not regulated at the transcriptional level but at the post-transcriptional level by phosphorylation by MAP kinases. MAPK dependent phosphorylation of Yan and Tel is the central mechanism that regulates the activity and stability of these transcriptional repressors [22–26] (reviewed in [27]). Specific phosphorylation events triggered by either ERK, JNK or p38 downregulate the transcriptional repressor function of *Drosophila* Yan or of vertebrate Tel, leading to their export out of the nucleus and to their degradation [22–26] (reviewed in [27]). Mutations that convert the serine and threonines residues normally phosphorylated by MAPK into non-phosphorylatable residues transform these factors into constitutively active repressors while mutations that convert them into phospho-mimetic residues promote degradation of these factors. The sea urchin Yan/Tel protein contains 3 canonical consensus MAPK phosphorylation sites PXS/TP located between the Sam domain and the Ets domain and in the C-terminal region plus one additional LSTP site that has been shown to be phosphorylated efficiently by ERK [39]. To investigate the roles of these potential phosphorylation sites in the regulation of Yan/Tel transcriptional activity and or/stability in vivo, we constructed a mutant Yan/Tel, Yan/Tel4A (Yan/Tel MAPK mutant), in which these putative MAPK phosphorylation sites were replaced by alanine (Fig 4A). Replacing the four putative MAPK consensus phosphorylation sites by an alanine increased the ability of Yan/Tel to repress *nodal* at blastula stage (absent in 58% of the embryos) and caused a partial radialization of the embryos at 36h. However, these embryos progressively recovered a dorsal-ventral polarity as shown by their pluteus-like morphology at 72 h.

We then examined carefully the sequence of Yan/Tel and noticed that in addition to MAPK sites, sea urchin Yan/Tel contains two motifs that are potentially phosphorylatable by the Proline-directed kinase GSK3 (Fig 4A). The first GSK3 phosphorylation motif conforms to the well-defined β-TRCP destruction box DSGXXS. β-TRCP is a F-box subunit of the SCF (SKP1-CUL-F-box) complex E3 ubiquitin ligase that targets several proteins for polyubiquitinylation and proteasomal degradation including Snail, β-Catenin, Emi and IKβ [40–43]. β-TRCP recognizes the doubly phosphorylated DpSGXXpS motif of target proteins and adds ubiquitin to a proximal upstream lysine. The second motif of Yan/Tel potentially phosphorylatable by GSK3, referred below as the Ser/Thr cluster, **S**SST**T**PSP**S**PP, lies at position 669–678, near the C terminal end of the protein, and may conform to the requirement of GSK3 for a phosphorylated Ser/Thr residue at position p+4 of the substrate. We therefore constructed a series of mutants carrying mutations in these sites alone or in combinations (Fig 4A). The β-TRCP mutant, DAGHSA, bears mutations in the two residues presumed to be phosphorylated by GSK3. The Yan/Tel “cluster” mutant, ASPTAPAPAP contains 4 mutations in the Ser/Thr -rich region of Yan/Tel. The Yan/Tel8A mutant corresponds to combinations of MAPK sites mutations and Cluster mutations while the Yan/Tel 10A mutant bears mutations in the MAPK, cluster and β-TRCP motifs. Finally, the Yan/Tel13A mutant carries mutations in the MAPK, cluster and β-TRCP motifs plus 3 additional mutations in SP sites that can potentially be phosphorylated by Proline directed kinases (Fig 4A)(see materials and methods). These phosphorylation mutants were overexpressed at the same concentration as the MAPK mutant and the effects on the morphology of the embryos and on the expression of marker genes were analyzed. Like mutation of the putative MAPK sites, mutation of the β-TRCP or cluster motifs affected the activity of Yan/Tel and caused a partial radialization suggesting that these sites participate to the regulation of the activity/stability of Yan/Tel (Fig 4B). However, none of these mutants alone was sufficient to repress *nodal* expression efficiently and at 72 hpf, most the injected larvae had recovered a normal pluteus morphology. Similarly, overexpression of Yan/Tel8A (mutations in the cluster+MAPK sites) caused a partial radialization and resulted in an incomplete loss of *nodal* expression (absent in 50% of the embryos). Injection of mRNA encoding Yan/Tel 10A and 13A caused the strongest effects. They suppressed *nodal* expression in most embryos (91% and 85% of the embryos, respectively) and abolished dorsal-ventral axis formation (Fig 4B and 4C and see below). These observations suggest that mutations of the MAPK, cluster region and β-TRCP phosphorylation sites have synergistic effects on the activity/stability of Yan/Tel and mutations of all three types of sites are required to convert Yan/Tel into a constitutively active repressor.

**Fig 5:**
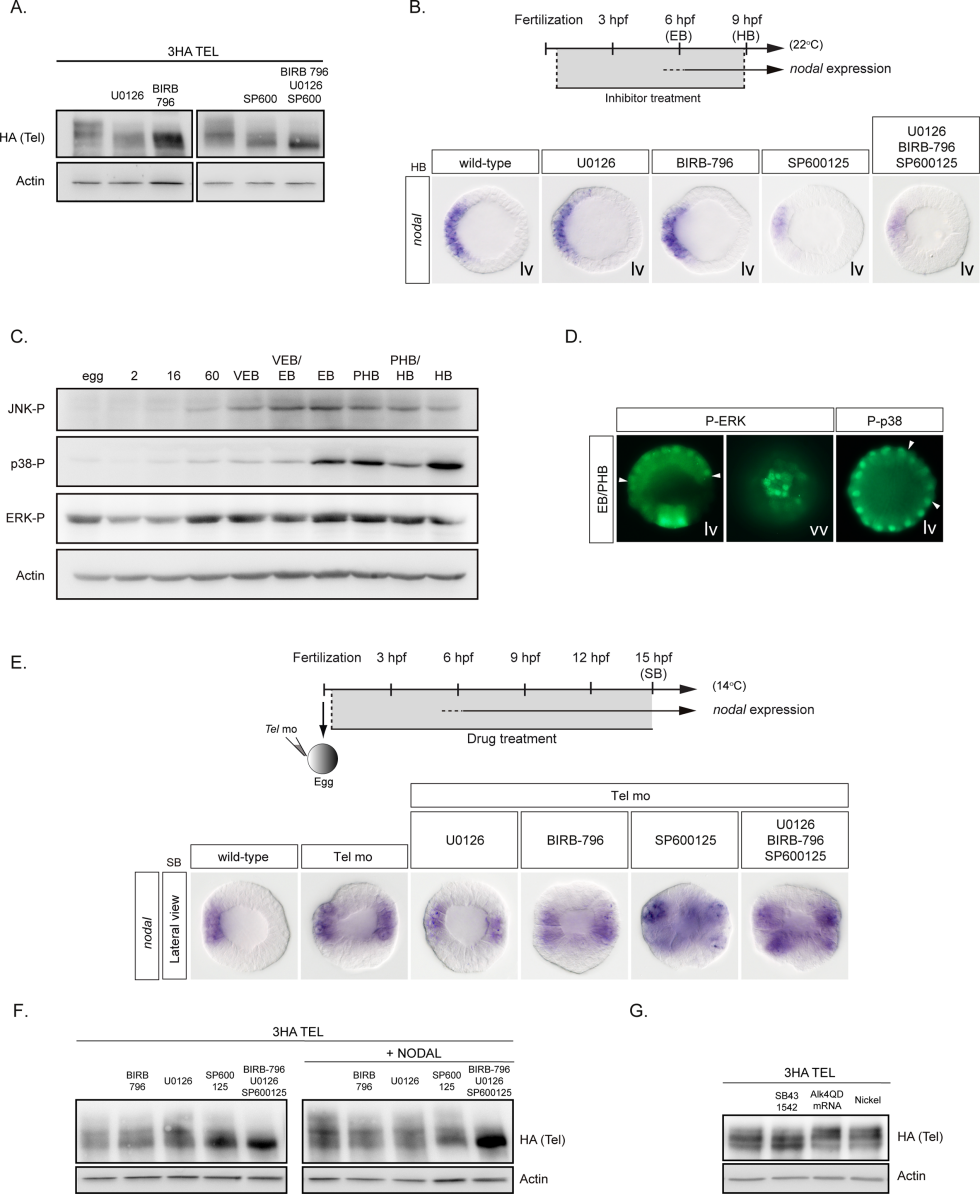
MAP kinases phosphorylate sea urchin Yan/Tel. A, Western blot against a wild type HA-tagged version of *yan/tel* in the presence or absence of the p38 inhibitor BIRB-796, the ERK inhibitor U0126, the JNK inhibitor Sp600125 or a combination of all three drugs. Wild-type Yan/Tel typically migrates as multiple bands with three to four migrating isoforms. Slower migrating isoforms are absent after treatment with the BIRB-796, U0126 or SP600125 drugs while wild type Yan/Tel migrates as a unique faster migrating isoform after simultaneous inhibition of all three MAP Kinases. B, p38, JNK and ERK are required for strong expression of *nodal*. Treatment with the JNK inhibitor SP600125 or combined inhibition of p38, ERK and JNK reduce although do not abolish the expression of *nodal*. Lv, lateral view. HB, hatching blastula, EB, early blastula. C, Western blot against p38, JNK and ERK phosphorylated forms during development. Note that these MAP kinases are upregulated between the 60-cell stage and the EB stage. 2, 16 and 60 refers to the number of cells; VEB, Very Early Blastula; EB, Early Blastula; PHB, Prehatching Blastula; HB, Hatching blastula. D, Fluorescent immunostaining against phosphorylated forms of ERK and p38. At early blastula stage, nuclear P-ERK is detected in ectodermal cells (arrowheads) and in the precursors of the skeletogenic mesoderm located at the vegetal pole, while nuclear P-p38 shows a broader distribution. The dorsal clearance of nuclear P-p38 staining is pointed by arrowheads. EB, Early Blastula; PHB, Prehatching Blastula. E, Epistasis experiments with Yan/Tel, p38, JNK and ERK. Similar to the inhibition of *yan/tel* function, simultaneous inhibition of p38, ERK or JNK and Yan/Tel results in a massive ectopic expression of *nodal*. SB, Swimming blastula stage. F, Western blot against the HA-tagged form of wild type Yan/Tel. Overexpression of Nodal mRNA enriches the slowest migrating isoform of wild type Yan/Tel suggesting that Nodal can promote the phosphorylation of Yan/Tel. This effect can be reversed by the addition of the MAP kinase inhibitors of p38, ERK or JNK. vv, vegetal view. lv, lateral view. In lateral views, animal is to the top. G, Western blot against the HA-tagged form of wild type Yan/Tel. Treatment with the Nodal receptor inhibitor SB431542 enriches the fast migrating Yan/Tel isoform. Reciprocally, overexpression of the mRNA encoding the activated Nodal receptor Alk4QD or treatment with nickel chloride (a treatment that phenocopies *nodal* overexpression) promotes phosphorylation of Yan/Tel as indicated by the enrichment of the slowest migrating isoform of Yan/Tel.

To analyze the effects of mutations in the MAPK, β*-*TRCP and cluster phosphorylation sites on the stability of Yan/Tel, we constructed tagged versions of wild-type and mutant Yan/Tel carrying three HA epitopes tag on the N-terminus. Having established that the presence of the HA tag did not alter the activity of the wild type Yan/Tel protein, we compared the effects of the various mutations on its phosphorylation state and stability. Wild type Yan/Tel typically migrated as multiple bands following SDS-PAGE with three to four slow migrating isoforms and a fourth barely detectable faster migrating isoform (see Fig 5A). Mutation of the β-TRCP degradation motif did not change significantly the migration of Yan/Tel but it resulted in a marked stabilization of the protein as judged by the much stronger intensity of the band of Yan/Tel β-TRCP mutant compared to wild type (Fig 4D). In contrast, replacing the four putative MAPK consensus phosphorylation sites by an alanine changed dramatically the mobility of Yan/Tel, which then migrated predominantly as a fast migrating isoform. This observation suggests that the slow migrating isoforms of Yan/Tel are isoforms phosphorylated by MAPKs. Mutation of the Ser/Thr cluster also resulted in a significant stabilization of the protein but interestingly, in this case, a fast migrating isoform was also predominant. Finally, a mutant form of Yan/Tel carrying mutations of the MAPK, Ser/Thr cluster and β-TRCP motifs showed a strong stabilization and migrated, like the cluster mutant, predominantly as a fast migrating, presumably unphosphorylated isoform (Fig 4D). Taken together these results show that combining mutations in the MAPK sites, Ser/Thr cluster and β-TRCP motif produce synergistic effects on the stability of Yan/Tel and that phosphorylation on several sites of Yan/Tel likely regulates its stability and/or activity.

Finally, we tested if random injection of *yan/tel13A* mRNA into one blastomere at the two-cell stage is sufficient to orient the dorsal-ventral axis. Indeed, in all the injected embryos (n = 15), *nodal* expression was found in a discrete region on the opposite side of the cells expressing *yan/tel13A* and the progeny of the injected blastomere later occupied a territory that precisely coincided with the dorsal ectoderm (Fig 4E).

Taken together these observations show that Yan/Tel acts as a major negative regulator of *nodal* expression. The stability of Yan/Tel is itself regulated by GSK3, which is active in the ectoderm and which targets Yan/Tel for degradation. Therefore, Yan/Tel may act at the crossroads of both animal-vegetal and dorsal-ventral patterning.

### MAPK and Nodal signaling modulate the activity/stability of Yan/Tel during D/V axis formation

Treatments with the MEK inhibitor U0126, or with the p38 inhibitor BIRB796 or with the JNK pathway inhibitor SP600125 had modest effects on the stability of Yan/Tel, although we noticed that the slowest migrating isoform of Yan was consistently reduced or missing following treatment with either of these inhibitors (Fig 5A). Strikingly, when used in combination, U0126, BIRB-796 and SP600125 increased the stability of Yan/Tel which then migrated predominantly as a fast migrating isoform. Western blot analysis confirmed that ERK, p38 and JNK are active in the early embryos (Fig 5C). Furthermore, immunostaining experiments revealed that, in addition to being present in the skeletogenic mesoderm located at the vegetal pole, activated forms of ERK are also present in the ectoderm at early blastula stage while p38 is more broadly active and detected in most nuclei with a shallow dorsal-ventral gradient (Fig 5D)[38, 44]. However, treatments from fertilization on with inhibitors of ERK, p38 or JNK reduced but did not suppress *nodal* expression (Fig 5B), the strongest effect being observed in the case of JNK inhibition. These observations are consistent with the idea that MAPK contribute to the regulation of *nodal* expression by phosphorylating and destabilizing Yan/Tel but that additional kinases are likely involved in the regulation of the activity/stability of this transcription factor. Finally, treatments with MAPK inhibitors alone or in combination did not suppress the ectopic expression of *nodal* observed in Yan/Tel morphants consistent with the idea that MAPK act upstream of Yan/Tel (Fig 5E).

A plethora of studies have documented that Activin, Nodal and TGF-ß signaling secondarily induce MAPK signaling [45–48]. We therefore tested if Nodal signaling impacts on phosphorylation of Yan/Tel by specific MAPK (Fig 5F). Overexpression of *nodal* mRNA, overexpression of a constitutively active Nodal receptor or treatment with nickel did not dramatically alter the stability of Yan/Tel but we noticed that they reproducibly promoted formation of the slowest migrating (presumably phosphorylated) isoforms of Yan/Tel (Fig 5F and 5G). All three MAPKs were apparently required for this effect (Fig 5F). In contrast treatment with the Nodal receptor inhibitor SB431542 appeared to favor formation of the faster migrating isoform of Yan/Tel, which likely represents the unphosphorylated isoform (Fig 5G). Although Nodal signaling did not appear to affect the overall stability of Yan/Tel, it did change its phosphorylation state. It is therefore possible that Nodal signaling impacts on MAPK signaling which in turn modifies the activity of Yan/Tel. These results are consistent with a model in which Nodal signaling acting through MAPK signaling, indirectly promotes phosphorylation and downregulation of Yan/Tel, thereby reinforcing *nodal* expression.

### GSK3 mediated phosphorylation of Yan/Tel regulates its stability

**Fig 6:**
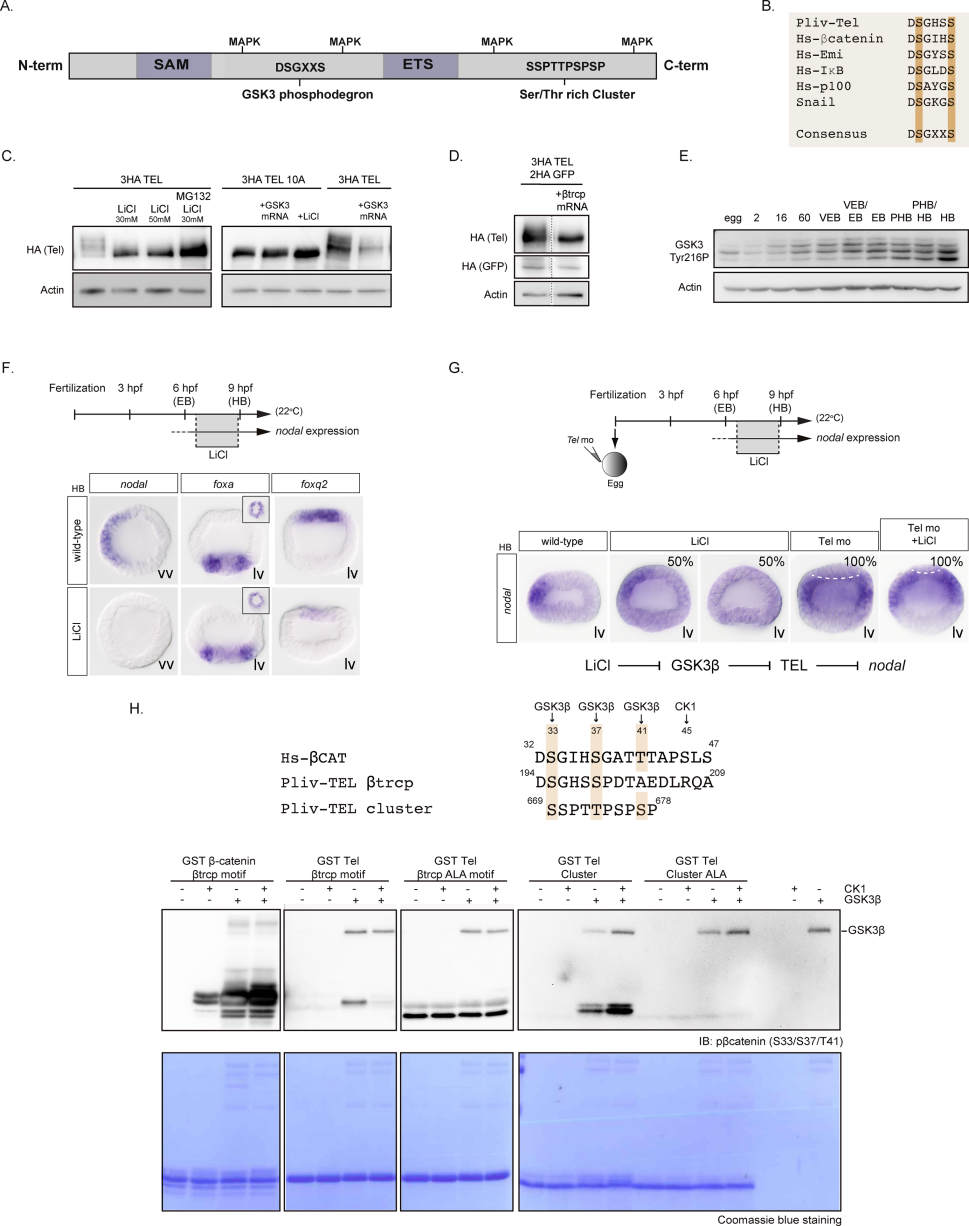
GSK3β phosphorylates sea urchin Yan/Tel and regulates its stability. A, Structure of Yan/Tel protein. B, Alignment showing conservation of the β-TRCP motif of sea urchin Yan/Tel and human β-catenin, Emi, IKβ, p100 and Snail proteins. Conserved phosphorylation sites are highlighted in orange. C, Western blot against the HA-tagged form of wild type or the phosphorylation mutant Yan/Tel (Yan/Tel10A). Wild-type Yan/Tel typically migrates as multiple bands with three to four migrating isoforms while the phosphorylation mutant Yan/Tel10A migrates as a unique and faster migrating isoform. Slower migrating isoforms of wild type Yan/Tel are absent after treatment with lithium, a GSK3β inhibitor. Note the enrichment of wild type Yan/Tel protein after treatment with the GSK3β inhibitor and the proteasome inhibitor MG132. Reciprocally, overexpression of GSK3β mRNA causes a strong reduction of the intensity of the signal of wild type Yan/Tel. The abundance of the unique and faster isoform observed after overexpression of the phosphorylation mutant Yan/Tel10A it is not altered by the presence of lithium or GSK3β mRNA. D, Western blot against the HA-tagged form of wild type Yan/Tel. Overexpression of mRNA encoding the β-TRCP ubiquitin ligase eliminates the slower phosphorylation isoforms of wild type Yan/Tel. HA-Tagged GFP mRNA was co-injected as a control. E, Western blot against GSK3-Tyr216 phosphorylated form during development. Note that GSK3 phosphorylated on Tyr216, which is the active form of this kinase, is upregulated during cleavage and peaks at the early blastula stage. 2, 16 and 60 refers to the number of cells; VEB, Very Early Blastula; EB, Early Blastula; PHB, Prehatching Blastula; HB, Hatching blastula. F, Short treatments with lithium abolish *nodal* expression. A short treatment with lithium at early blastula stage is sufficient to completely eliminate *nodal* expression and to dramatically reduce *foxq2* expression, without expanding the expression of the endodermal marker *foxa*. vv, vegetal view. lv, lateral view. The upper panels show vegetal views of the same embryo. In lateral views, animal is to the top. G, Epistasis experiments with Yan/Tel and short treatment with lithium. In this experiment, a short treatment with lithium eliminated *nodal* expression in 50% of the treated embryos. In contrast, similar to the ectopic expression of *nodal* observed after inhibition of *yan/tel* function, simultaneous inhibition of GSK3β and Yan/Tel resulted in a massive ectopic expression of *nodal* in 100% of the observed embryos. HB, Hatching blastula stage. lv, lateral view. Animal is to the top. H, GSK3β phosphorylates Yan/Tel in vitro. Alignment of the sea urchin Yan/Tel β-TRCP and cluster domains and the β-TRCP domain of human β-catenin. Conserved positions of putative phosphorylation sites are highlighted in brown. Western blot of a GSK3β-CK1 in vitro kinase assay shows phosphorylation by the Kinase GSK3β of the wild type β-TRCP and cluster sea urchin Yan/Tel GST–fused peptides but not of the alanine mutated GST peptides. A GST-peptide of the β-TRCP domain of human β-catenin was used as a control.

Since Yan/Tel contains a β-TRCP destruction motif (Fig 6A and 6B) that could potentially be phosphorylated by the Proline directed kinase GSK3, we tested the effects of blocking GSK3 on the activity/stability of Yan/Tel. Treatment of sea urchin embryos with increasing concentrations of lithium, a potent GSK3 inhibitor, resulted in a marked and dose-dependent increase in the stability of HA-tagged Yan/Tel (Fig 6C). Co-treatment with lithium and MG132, a proteasome inhibitor, caused a synergistic accumulation of Yan/Tel. Remarkably, treatment with lithium also dramatically altered the migration pattern of sea urchin Yan/Tel protein. While in control embryos the Yan/Tel protein migrated as four distinct isoforms on an SDS-PAGE gel, following lithium treatment Yan/Tel migrated predominantly as the faster migrating isoform (Fig 6C), which we presume is a non-phosphorylated form of this factor. Similarly, overexpression of β-TRCP mRNA resulted in Yan/Tel protein migrating predominantly as a fast migrating non-phosphorylated isoform consistent with the idea that the slowest migrating isoforms of Yan/Tel are isoforms phosphorylated on the β-TRCP motif that are recognized and degraded by the ubiquitin ligase β-TRCP (Fig 6D). To confirm that GSK3 regulates the stability of Yan/Tel, we overexpresse*d* sea urchin GSK3β with either wild type Yan/Tel or with the Yan/Tel10A mutant which lacks the MAPK, β-TRCP and cluster phosphorylation motifs (Fig 6C). Overexpression of GSK3 significantly reduced the level of wild type Yan/Tel protein but had no detectable effect on the stability of Yan 10A reinforcing the idea that GSK3 phosphorylates Yan/Tel and targets it for degradation. In agreement with the western blot analysis, which indicated that inhibition of GSK3 stabilizes Yan/Tel, a 3h treatment with lithium performed at early blastula stage (Fig 6F), when GSK3 is active (Fig 6E), was sufficient to extinguish *nodal* expression (Fig 6F). This extinction of *nodal* caused by lithium occurred without any concomitant expansion of *foxA* expression indicating that it was not caused by an indirect effect such as a change in specification of the ectoderm but more likely by a more direct effect of inhibition of GSK3 on Yan/Tel stability. Finally, treatments with lithium did not block the ectopic expression of *nodal* observed in Yan/Tel morphants consistent with the idea that GSK3 acts upstream of Yan/Tel (Fig 6G).

We conclude that phosphorylation by GSK3 and MAPK followed by proteasome-mediated proteolysis are key processes that regulate the stability and/or activity of Yan/Tel. However, it should be noted that the activity of the overexpressed Yan/Tel mutants as measured by their impact on *nodal* expression (Fig 4C) do not correlate perfectly with the effects of these mutations on the stability of Yan/Tel, with GSK3 preferentially affecting the stability and MAPKs affecting more the activity of Yan/Tel (Fig 5A; Fig 6C). This highlights the complex nature of the regulation of Yan/Tel by these kinases.

### Yan/Tel is phosphorylated in vitro by GSK3 on two different regions

We have shown that sea urchin Yan/Tel contains at least two consensus sites for phosphorylation by GSK3, that inhibition of GSK3 affects the stability of Yan/Tel and that mutation of these sites changes the activity of the protein. However, evidence that GSK3 can phosphorylate these sites was still missing. To further examine if sea urchin Yan/Tel can serve as a substrate for GSK3, we set up an in vitro phosphorylation assay to detect phosphorylation of Yan/Tel at Ser 195 and Ser 198. Since no phospho-specific antibody recognizing the phosphorylated motif of sea urchin Yan/Tel was available we used an alternative antibody. We reasoned first that the β-TRCP recognition motif of sea urchin Yan/Tel (D**S**GHS**S**PDT) shares similarity with the β-TRCP motif of human β-catenin D**S**GIH**S**GAT**T** (Fig 6H). Therefore, an antibody against the doubly phosphorylated DpSGIHpS motif of human βcatenin may recognize the DpSGHSpS motif of sea urchin Yan/Tel. We therefore produced and purified a glutathione–S-transferase (GST)-fused Yan/Tel peptide corresponding to the β-TRCP motif of the sea urchin protein. We used as control a GST-fused β-catenin peptide carrying the β-TRCP recognition motif of human β-catenin. These peptides were used as substrates in a kinase reaction then the reaction products were separated by SDS PAGE, blotted and immunodetection was carried out with the anti-phospho (Ser33/Ser37/Thr41)-human β-catenin. Since GSK3 requires a priming phosphorylation by GSK3 to phosphorylate β-catenin, we performed kinase reactions either without or with purified Casein kinase alone, GSK3 alone or with the two kinases in combination. Western blot analysis revealed that the anti phospho-ß catenin antibody indeed recognizes the β-TRCP motif phosphorylated by GSK3 (Fig 6H). However, unlike β-catenin, which requires pre-phosphorylation by Casein kinase, sea urchin Yan/Tel does not require Casein kinase to be phosphorylated by GSK3. Intriguingly, the addition of Casein kinase actually interfered with phosphorylation of the peptide carrying the sea urchin Yan/Tel β-TRCP degradation motif. No phosphorylation was detected in control reactions in which the substrate was the non phosphorylatable Alanine mutant β-TRCP degradation box DAGHSAPDT indicating that Yan/Tel is phosphorylated most likely directly by GSK3 at positions 195 and 198 (Fig 6H).

Finally, we also speculated that the antibody against the triply phosphorylated ß-catenin (Ser33/Ser37/Thr41) may recognize the triply phosphorylated Ser/Thr cluster of sea urchin Yan/Tel **S**SST**T**PSP**S**PP. Indeed, western blot analysis detected a band that corresponded to the Ser/Thr rich cluster of Yan/Tel phosphorylated by GSK3 (Fig 6H). This band was not detected when a non-phosphorylatable Alanine mutant Ser/Thr cluster peptide was used.

To summarize, we have shown that the β-TRCP destruction box and the Ser/Thr rich cluster of sea urchin Yan/Tel are consensus GSK3 sites, that inhibition of GSK3 alters the stability of Yan/Tel, that mutation of these sites changes the stability and/or the activity of Yan/Tel and that these sites can be phosphorylated by purified GSK3 further reinforcing the conclusion that GSK3 is a key regulator of the stability and activity of sea urchin Yan/Tel.

### Panda acts upstream of Yan/Tel to restrict *nodal* expression

**Fig 7:**
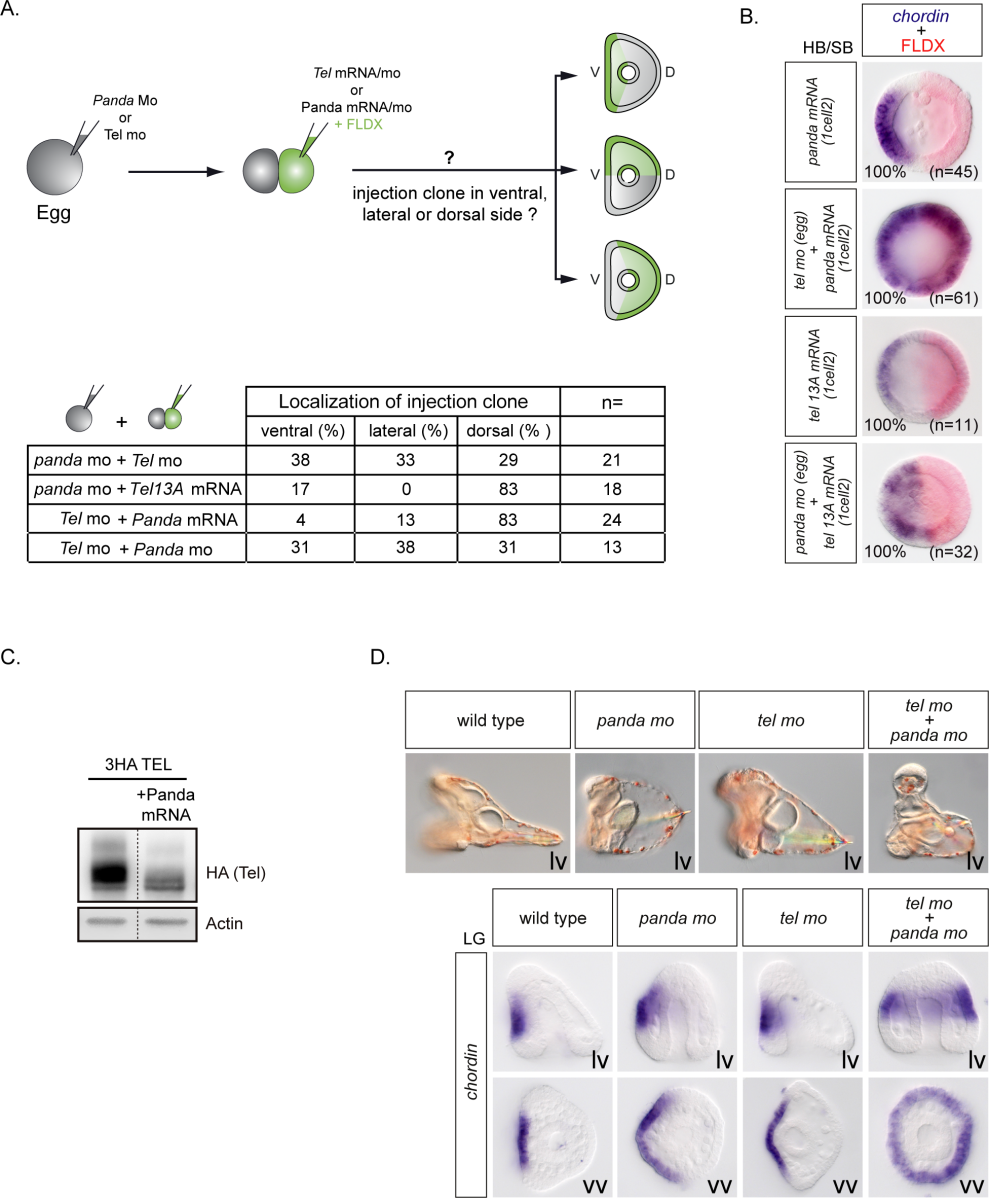
Epistasis experiments with Panda and Yan/Tel. A, Overexpression of *yan/tel* mRNA or *panda* mRNA into one cell at the two-cell stage orients the axis even in the absence of Panda or Tel/Yan, respectively. B, *panda* mRNA overexpression into one cell at the two-cell stage (FLDX clone developed in red) does not restrict early *nodal* expression (blue) to the ventral side in the absence of Yan/Tel. Instead, Yan/Tel mRNA overexpression into one cell at the two-cell stage (FLDX clone developed in red) confine *nodal* expression (blue) to the ventral side even in the absence of Panda. Vegetal views, ventral to the left. C, Western blot against the HA-tagged form of wild type Yan/Tel. Overexpression of the mRNA encoding Panda eliminates the slower phosphorylation isoforms of wild type Yan/Tel. D, Embryos injected with suboptimal doses of Panda or Yan/Tel morpholinos develop into pluteus larvae, while double Panda + Yan/Tel morphants appear partially ventralized and show radial *chordin* expression at late gastrula stage (LG). lv, Lateral view, ventral to the left. vv, vegetal view.

The similarity of the phenotypes caused by inactivation of Panda and Yan/Tel strongly suggested the possibility that Yan/Tel may act downstream of Panda. According to this model, Panda signaling may regulate *nodal* expression by inhibiting phosphorylation of Yan/Tel and stabilizing this factor. The fact that both Yan/Tel and Panda are required early to restrict *nodal* expression supports this idea (Fig 3A see Haillot et al. 2015). Furthermore, not only inactivation of either Yan/Tel or Panda causes ectopic expression of *nodal* but they both appear to do so in a cell-autonomous manner. To test if Panda and Yan Tel act in the same pathway and if Panda acts through stabilization of Yan/Tel, we tested the ability of Panda to orient the D/V axis in the absence of Yan/Tel and reciprocally, the ability of Yan/Tel to orient the axis in the absence of Panda. Fertilized eggs were injected either with the Yan/Tel or Panda morpholinos and at the 2-cell stage, *panda* mRNA or constitutively active *yan/tel* mRNA was injected into one blastomere at the 2-cell stage. *nodal* expression was then analyzed at early blastula stage and the position of the territory derived from the injected clone was later recorded at prism stage (Fig 7A). In Yan morphants at prism stage, the progeny of the clones injected with Panda mRNA was found predominantly on the dorsal side indicating that Panda can orient the D/V axis of Yan morphants. However, while in control embryos local overexpression of *panda* mRNA efficiently oriented the D/V axis and efficiently restricted *nodal* expression at early blastula stage, strikingly, in *yan/tel* morphants injected with Panda mRNA, *nodal* remained expressed ubiquitously at early blastula stage (Fig 7B). That Yan morphants injected with Panda mRNA into one blastomere failed to restrict *nodal* expression at early stages strongly suggests that Yan is epistatic to Panda, or in other words, that Yan acts downstream of Panda. Consistent with this idea, local overexpression of activated *yan/tel* efficiently restricted *nodal* expression and oriented the D/V axis in *panda* morphants (Fig 7A and 7B). To further test if Yan and Panda act in the same pathway during D/V axis formation, we looked for synergistic effects. Injection of suboptimal doses of either the Yan or the Panda morpholino resulted in a spatially restricted expression of the nodal target gene *chordin* at gastrula stage. In contrast, coinjection of the Yan and Panda morpholinos at these suboptimal doses caused a dramatic expansion of *chordin* expression and resulted in a strong ventralization (Fig 7D). These results strongly suggest that Yan/Tel and Panda likely work in the same pathway and synergize to restrict *nodal* expression. Finally, we tested if overexpression of Panda affects the phosphorylation of Yan/Tel by western blot. Overexpression of Panda caused Yan/Tel to migrate predominantly as a fast migrating (presumably stabilized) isoform, consistent with the idea that Panda acts at least in part by regulating the phosphorylation state and therefore the stability and/or the activity of the transcription factor Yan/Tel (Fig 7C).

In conclusion, we have identified Yan/Tel as a new central negative regulator of the early expression of *nodal* and possibly as a downstream effector of the maternal determinant Panda. Taken together these data suggest that the stability of Yan/Tel is regulated by the combined activities of MAP Kinases and GSK3, and possibly of Nodal itself, and that these factors cooperate to target this repressor for degradation by a β-TRCP/proteasome degradation pathway. Finally, our data suggest a model in which Panda acts upstream of Yan/Tel to restrict *nodal* expression, possibly by antagonizing phosphorylation and degradation of Yan/Tel on the dorsal side. By integrating information along the animal-vegetal and dorsal ventral axis, Yan/Tel may therefore act as a factor that coordinates patterning along these two orthogonal patterning systems.

## Discussion

### Yan/Tel as a key maternal regulator of *nodal* expression

Recent studies on the promoter region of the sea urchin *nodal* gene have started to identify maternal transcription factors required for high level of *nodal* expression [4, 49, 50]. However, while these studies successfully identified binding sites for positive regulators of *nodal* expression including SoxB1, bZIP, and Oct factors, they failed to identify any spatial regulator of *nodal* expression. In this study, we have identified the ETS containing transcriptional repressor Yan/Tel as a key spatial regulator of *nodal* expression and as a target of MAP kinases and GSK3 in *Paracentrotus lividus* embryos. We found that inactivation of Yan/Tel causes ectopic expression of *nodal*, a phenotype largely similar to that caused by the loss of the maternal determinant Panda. Indeed, we discovered that Yan/Tel is epistatic to Panda strongly suggesting that Panda acts at least in part by stabilizing Yan/Tel on the dorsal side. Furthermore, we discovered that the activity and/or stability of Yan/Tel is negatively regulated by the combined activities of MAP kinases and GSK3 and possibly of Nodal itself. Taken together, these findings shed light on the mechanisms regulating the early expression of *nodal* at the onset of development and suggest that Yan/Tel acts as an integrator of patterning signals along the animal-vegetal and dorsal-ventral axes.

### Panda as an upstream modulator of Yan/Tel activity/stability

The key observation at the basis of this study was that downregulation of maternal but not zygotic Yan/Tel function by injection of morpholino oligonucleotides caused a massive but transient ectopic expression of *nodal* in the dorsal ectoderm, largely mimicking the effects of inactivating the maternal factor Panda and partially mimicking the effects of inactivating Lefty. The maternal TGF-β ligand Panda was recently proposed to control dorsal-ventral axis formation by restricting the early expression of *nodal* [15]. Intriguingly, the phenotypes resulting from inactivation of maternal Panda function are very similar to those resulting from inactivation of Yan/Tel strongly suggesting that both genes may work in the same pathway. In both Panda and Yan/Tel morphants, *nodal* is massively ectopically expressed throughout most of the ectoderm resulting in full radialization of the ectoderm and endomesoderm and causing ectopic expression of ventral marker genes during gastrulation. Although the phenotypes of *yan/tel* and *panda* morphants are strikingly similar, there are however important differences between the two factors. In the case of Panda, in situ hybridization revealed a D/V asymmetry of maternally deposited *panda* mRNA and rescue experiments confirmed that the activity of Panda is likely spatially restricted in the embryo. Therefore, Panda clearly has some properties of a maternal determinant of the D/V axis. In contrast the *yan/tel* maternal RNA is uniformly distributed in the egg and early embryo and rescue experiment indicate that *yan/tel* mRNA does not need to be spatially restricted to rescue the D/V axis of *yan/tel* morphants.

**Fig 8:**
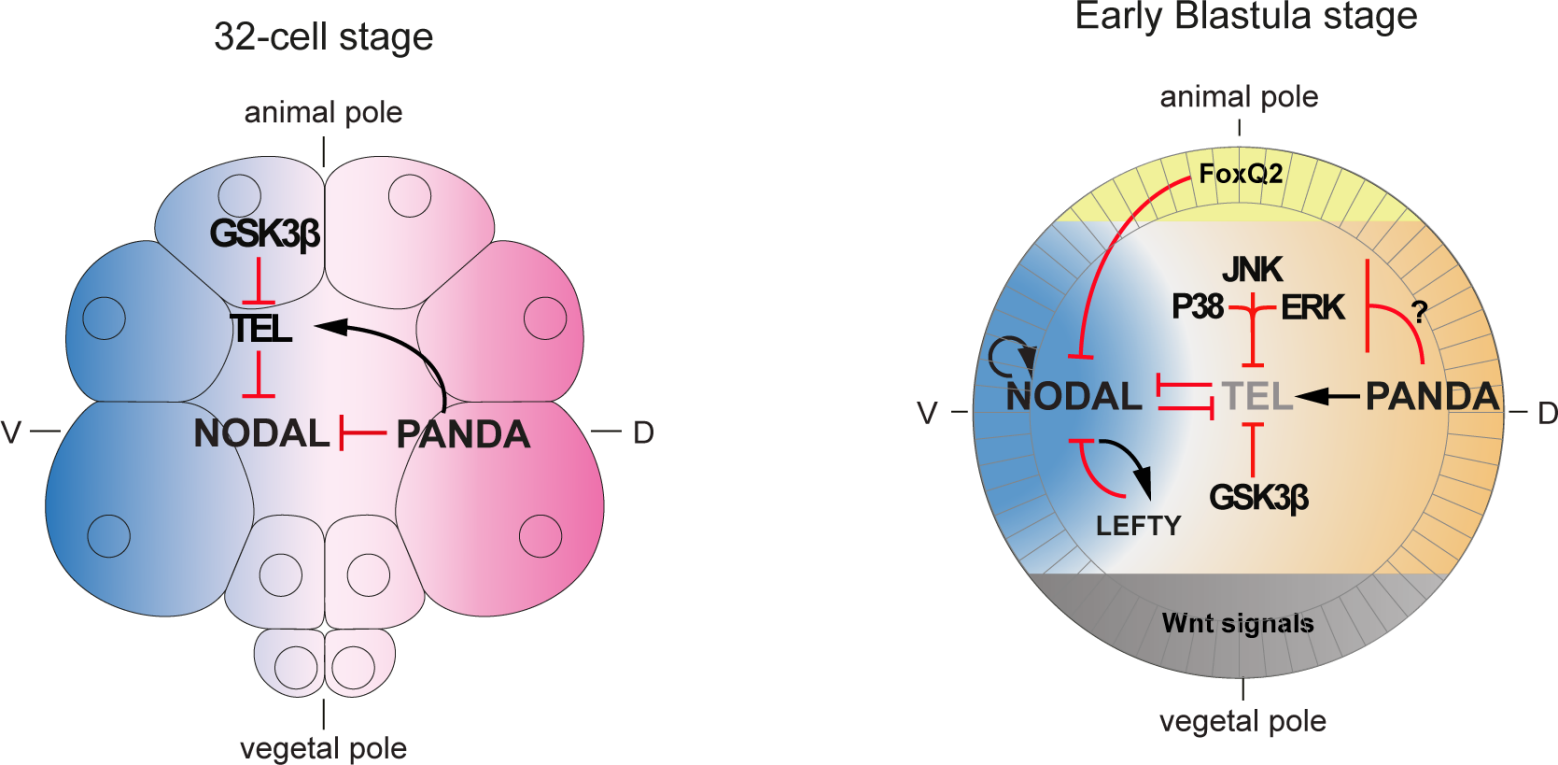
Model of the regulation of *nodal* expression by GSK3, Yan/Tel and Panda. Model of the regulation of *nodal* expression by the maternal factors Yan/Tel and Panda. Starting at the 32-cell stage GSK3 activity in the animal hemisphere starts to target Yan/Tel for degradation, thereby releasing the repression of Yan/Tel on *nodal* expression and allowing *nodal* expression to be initiated. On the dorsal side of the embryo, Panda creates the asymmetry of *nodal* expression by antagonizing *nodal* expression by a mechanism that is not elucidated but that may rely on the function of Yan/Tel. At early blastula stage, *nodal* expression in the animal pole is repressed by the presence of FoxQ2. In the rest of the ectoderm, MAP kinases, GSK3 and possibly Nodal signaling contribute to promote *nodal* expression by promoting phosphorylation and degradation of Yan/Tel. On the contrary, Panda signaling on the dorsal side may prevent degradation of Yan/Tel contributing to repress nodal expression on the dorsal side.

According to one scenario, Panda and Yan/Tel may act independently, i.e. in parallel, to restrict *nodal* expression. Panda may first act to break the radial symmetry and to restrict *nodal* expression and the MAP kinase pathways and Yan/Tel may then act to maintain this asymmetry. Alternatively, Panda and Yan/Tel may act in a coordinated manner in the same pathway, as suggested by the similarity of the phenotypes caused by inactivation of either gene. According to this hypothesis, Panda may provide the initial spatial cue that restricts *nodal* by downregulating the activity of MAPK, thereby reinforcing the repressive effect of Yan/Tel on *nodal* on the presumptive dorsal side. In this study, we have provided several lines of evidence suggesting that Panda and Yan/Tel indeed likely function in the same pathway, First, we have shown that inactivation of Yan or Panda by morpholino injection caused a strikingly similar phenotype. Second, we have shown that Panda requires Yan function to restrict *nodal* expression while a constitutively active Yan does not require Panda function to do so. Third, we have shown that Panda and Yan strongly synergize during D/V axis formation consistent with the idea that these genes likely act in the same pathway and are linked by a functional or temporal hierarchy. Finally, we have provided biochemical evidence suggesting that overexpression of Panda causes accumulation of a predominantly non-phosphorylated, and presumably stabilized, form of Yan/Tel. Therefore, taken collectively, these findings strongly suggest a model of symmetry breaking based on the stabilization of Yan/Tel by Panda on the presumptive dorsal side. Indeed, all the evidence cited above are indirect and a definitive proof that Panda stabilizes Yan/Tel and or modulates its activity on the presumptive dorsal side will require additional studies. Nevertheless, we have identified Yan/Tel as a novel and essential regulator of *nodal* expression acting downstream (temporally or functionally) of Panda (Fig 8).

### Conservation of the mechanisms responsible for establishment of the D/V axis

It is presently unclear if the mechanisms that we have described and that implicate the maternal TGF beta Panda and the ETS domain transcription factor Yan/Tel are conserved in other species of sea urchin. We note however, that the *panda* and *yan*/*tel* genes are indeed present in the genomes of all sea urchin species that we have examined suggesting that this may be the case. Furthermore, the MAPK and GSK3 phosphorylation sites of Yan/Tel that we have identified in the protein from *Paracentrotus* are very well conserved between *P*. *lividus* and in *S*. *purpuratus* suggesting that the protein is submitted to the same regulation in both species. While Panda and Yan/Tel have not so far been implicated in D/V axis formation in *S*. *purpuratus*, early asymmetries in the distribution of mitochondria have been proposed to provide a spatial cue in the process leading to specification of the secondary axis [16, 51, 52]. Furthermore, in *S*. *purpuratus*, the position of the D/V axis has been shown to be correlated with the plane of first cleavage, a feature that has so far been observed only in that species [53]. Although gradients of mitochondrial activity have been described in *P*. *lividus* eggs and embryos, we have so far been unable to obtain evidence for a role of redox gradients or of hypoxia in establishment of the D/V axis in the Mediterranean sea urchin. It is therefore formally possible that different mechanisms operate in different species of echinoderms to establish the D/V axis and that *S*. *purpuratus* embryos may rely on a mechanism based redox gradients while *P*. *lividus* rely instead on Panda and Yan/Tel to set up the secondary axis. To clarify this question and to test if Panda and Yan/Tel are required in other species of sea urchin for D/V axis establishment, we have recently initiated a comparative study of the genes regulating the spatial restriction of *nodal* expression in different sea urchin species.

### Transient nature of the Yan and Panda loss-of-function phenotypes

Despite the massive ectopic expression of *nodal* during early stages, and the continued repression of dorsal marker genes until late in gastrulation, both *panda* morphants and *yan/tel* morphants progressively recover a spatially restricted expression of *nodal* and of dorsal marker genes between 36 and 72 h after fertilization so that on the third day of development, these embryos do not show the strong radialization that could have been anticipated from the observation of the same embryos at earlier stages. This progressive recovery of D/V polarity of *yan/tel* morphants raises a concern regarding the effects of the morpholino since it could be argued that injection of the morpholino might simply delay development and slow down establishment of the D/V axis. This is undoubtedly not the case. *yan*/*tel* morphants, as well as *panda* morphants, display a very specific and characteristic set of morphological and molecular phenotypes that are caused by the failure of a key and early step in the process of D/V axis specification: the early spatial restriction of *nodal* expression. Both *panda* and *yan*/*tel* morphants display a massive ectopic expression of *nodal* and Nodal target genes that starts at the 60-cell stage and lasts until late in gastrulation. Both *panda* and *yan*/*tel* morphants remain fully radialized up to the late gastrula stage and both adopt the typical morphology of embryos partially ventralized after treatment with recombinant Nodal protein. In conclusion, *panda* and *yan*/*tel* morphants are strongly ventralized, but this ventralization is not maintained during gastrulation probably because the maternal proteins involved in their function progressively disappear from the embryo allowing compensatory zygotic mechanisms to operate and the embryo to regulate.

### Regulation of Yan/Tel stability by GSK3 and β-TRCP

While searching for sites that may be phosphorylated by MAPK, we discovered that the sequence of sea urchin Yan/Tel contains at least two regions that may be phosphorylated by GSK3, which targets several proteins for degradation including β-catenin and Snail. The first motif DSGHSS conforms to the degradation box recognized by the E3 Ubiquitin ligase β-TRCP that recognizes the phosphorylated residues within the motif DpSGX_(1–4)_pS and promotes ubiquitinylation by the Skp1-Cul1-Fbox complex. The second putative GSK3 phosphorylation motif is a short region rich in serines, threonines and prolines and is located in the C-terminal region.

Although there is no strict consensus for phosphorylation by GSK3, it has been shown that GSK3 usually requires a priming phosphorylation for processive phosphorylation [54] and many GSK3 substrates conform to the sequence S/TXXXpS. Both β-catenin and Snail contain a DSGXXS motif immediately followed by a XXXS/TXXXS/T GSK3 phosphorylation sequence that is crucial for their regulation by this kinase. Intriguingly, the sea urchin Yan/Tel phosphodegron DSGXXS is immediately followed by a PDTAED motif, which does not resemble a canonical GSK3 site. It is therefore unclear if there is a priming kinase that phosphorylates Yan/Tel before GSK3 can phosphorylate the phosphodegron and if this phosphodegron is phosphorylated as efficiently as β-catenin and Snail. Nevertheless, we have provided several lines of evidence to support the idea that GSK3 is a key regulator of Yan/Tel activity/stability and that its β-TRCP motif is indeed phosphorylated by GSK3. First, we have shown that inhibition of GSK3 caused a dose dependent stabilization of Yan/Tel. Second, we have shown that, in vivo, overexpression of GSK3 strongly destabilized wild type Yan/Tel but did not affect the stability of a Yan13A, a phosphorylation mutant of Yan/Tel. Third, we have shown that mutation of the β-TRCP and S/T rich cluster motifs markedly stabilizes Yan/Tel. Finally, we have shown that both the β-TRCP motif and the S/T rich cluster are phosphorylated in vitro by purified GSK3. Therefore, Yan/Tel can be added to the list of bona fide GSK3 substrates in the sea urchin. Intriguingly, neither Yan from *Drosophila* nor Tel from vertebrates contain a β-TRCP motif in their sequence. Indeed, in the fly or in vertebrates, phosphorylation by MAP kinases, not GSK3, is the main mode of regulation of Yan or Tel and both Yan and Tel have been shown to be degraded primarily following ubiquitination by the ubiquitin conjugating enzyme FBL6 [55], not by β-TRCP. The regulation of sea urchin Yan/Tel by a GSK3/β-TRCP pathway therefore appears as a novel feature of this ETS family member. The regulation of sea urchin Yan/Tel by a GSK3/β-TRCP pathway may be linked to the very early function of Yan/Tel in specification of the dorsal-ventral axis of the sea urchin embryo and to its role as a coordinator of the A/V and D/V patterning systems.

### Similarities between the regulation of Yan/Tel and the regulation of β-catenin stability

The finding that Yan/Tel is targeted for degradation by a GSK3/β-TRCP pathway, like β-catenin, is particularly interesting. β-catenin turn-over has long been known to be regulated by GSK3–mediated proteolysis [56]. GSK3 is part of a multiprotein degradation complex that involves APC, Axin and Casein kinase. In the absence of Wnt, Casein kinase phosphorylates β-catenin and primes it for phosphorylation by GSK3. Phosphorylated β-catenin is then recognized by the β-TRCP ubiquitin ligase, which targets it to degradation by the proteasome. In the presence of Wnt ligands, the function of the destruction complex is inhibited and β-catenin levels increase allowing it to translocate into the nucleus where it interacts with TCF to regulate target genes. In the sea urchin embryo, β-catenin is highly unstable in the animal half blastomeres and accumulates in the nuclei of blastomeres located at the vegetal pole of the embryo and fated to become endoderm and mesoderm [57–59]. Although the spatial distribution of maternal Yan/Tel in the early embryo remains to be determined, it is reasonable to think that Yan/Tel is subject to a similar regulation i.e. that it is targeted for degradation by GSK3 in the animal blastomeres and stabilized in the vegetal pole blastomeres where it represses transcription of target genes including *nodal*. One difference however, is that phosphorylation of sea urchin Yan/Tel does not appear to require a priming phosphorylation by Casein kinase, and therefore the processivity of the phosphorylation of Yan/Tel by GSK3 may not be as high as that of β-catenin. Furthermore, zygotic *yan/tel* mRNA, accumulates in precursors of the endoderm and mesoderm that express Wnt ligands such as Wnt1, Wnt8 or Wnt6 and that do not express *nodal* [60–62]. It is therefore likely that canonical Wnt signaling in the vegetal pole region contributes to repress *nodal* expression in the endomesoderm by protecting Yan/Tel from GSK3-mediated proteolysis. Consistent with this idea, it has been reported that *nodal* is ectopically expressed in the vegetal pole region of Wnt1morphants[62]. It will be interesting in the future to test if Wnt ligands directly contribute to the stability of Yan/Tel and to further investigate if Yan/Tel is involved in the crosstalk between MAPK and Wnt signaling. Also, it will be interesting to determine if proteins such as Axin and APC participate to the GSK3-mediated regulation of Yan/Tel stability.

### Yan/Tel as a factor acting at the intersection of animal-vegetal and dorsal-ventral patterning

In this study, we discovered that Yan/Tel is a key regulator of D/V patterning and that its activity/stability is regulated by GSK3, a key regulator of patterning along the animal vegetal axis. Since GSK3 plays a central role in patterning along the animal vegetal axis by negatively regulating the stability of β-catenin in the ectoderm, our finding that Yan/Tel stability is regulated by GSK3 strongly suggests a mechanism by which Yan/Tel may integrate signals involved in patterning along the D/V and AP axis. While destabilizing β-catenin in the animal hemisphere to restrict formation of the endoderm to the vegetal pole, GSK3 may simultaneously destabilize Yan/Tel and allow the initiation of *nodal* expression during early cleavage. Our finding that Yan/Tel is also regulated by MAPK and the results of a recent study on the restriction of animal pole fate by Wnt/JNK signaling further suggest that, starting at 60 cell stage, a second interaction between Wnt signaling and Yan/Tel may occur [19]. Starting at 60-cell stage, early Wnt/β-catenin signaling initiates a cascade of signaling events mediated by Wnt1 and Wnt8 [19, 61] that activates Fz5/8 /JNK signaling in the ectoderm and restricts the animal pole domain. These findings raise the possibility that early Wnt/β-catenin signaling in the vegetal pole activates JNK signaling in the overlying ectoderm and JNK in turn may promote phosphorylation and degradation of Yan/Tel, thereby allowing *nodal* expression (Fig 8). Our finding that *nodal* expression is attenuated when JNK signaling is inhibited support this model. The finding that ERK is also activated in the ectoderm during this period suggests that additional MAPK dependent signaling events may impact on Yan/Tel stability to promote *nodal* expression.

The model presented in Fig 8 attempts to integrate our data on the regulation of Yan/Tel by GSK3 and MAPKs and by the maternal TGF-β Panda. According to this model, *nodal* is initially broadly expressed in the presumptive ectoderm at 32-cell stage by the activity of broadly distributed transcriptional activators such as SoxB1 and Oct1/2 as well as by the activity of GSK3, which inactivates Yan/Tel in the presumptive ectoderm. In this model, GSK3 plays an essential role on *nodal* expression by releasing the repressive effect of Yan/Tel but it does not provide the initial spatial cues that restricts *nodal* expression to the ventral side. Instead, the spatial restriction of *nodal* depends on the spatially restricted activity of the maternal TGF-β ligand Panda, which breaks the radial symmetry and starts to restrict *nodal* expression by a mechanism that requires the activity of the transcriptional repressor Yan/Tel. Although the mechanism by which Panda antagonizes Yan/Tel is likely indirect and not well understood, we propose that Panda modulates the activity or prevents degradation of the transcription factor Yan/Tel on the presumptive dorsal side. Then, at the early blastula stage, MAPKs signaling in the animal blastomeres further cooperates with GSK3 signaling to promote *nodal* expression through degradation of Yan/Tel. Nodal promotes its own expression and induces *lefty* expression and long-range inhibition by Lefty, together with Nodal autoactivation and repression by FoxQ2 in the animal pole domain, contributes to restrict *nodal* expression to the presumptive ventral ectoderm. Finally, signaling from the Alk4/5/7 Nodal receptor on the ventral territory may also contribute to maintain a high level of *nodal* expression in this territory possibly by promoting phosphorylation and destabilization of residual maternal Yan/Tel protein. A central and novel feature of this model is the MAP-kinase/GSK3/ß-TRCP /Yan/Tel double repression mechanism of *nodal* regulation, which is formally similar to the double negative gate that controls the PMC gene regulatory network.

In conclusion, this work identified Yan/Tel as a novel key maternal regulator of dorsal-ventral axis. Yan/Tel acts as a repressor of *nodal* expression whose expression activity/stability is positively regulated by GSK3, MAPKs and ß-TRCP and likely negatively regulated by Panda. We propose that phosphorylation of Yan/Tel triggers destruction of this maternal repressor in the animal blastomeres, which in turn allows *nodal* expression to be initiated in the presumptive ectoderm of the zygote. Nodal signaling may further reinforce MAPK signaling and contribute to downregulate Yan/Tel in the presumptive ventral ectoderm. We have therefore uncovered an essential new function for GSK3 in patterning of the sea urchin embryo: not only GSK3 controls the differential stability of β-catenin along the A/V axis [59, 63], but it simultaneously controls the expression of Nodal, a central regulator of patterning along the D/V axis. Therefore, this study uncovers a key interaction between the Gene Regulatory Networks responsible for patterning of the endomesoderm and ectoderm and sheds light on the mechanisms that coordinate patterning along the primary and secondary axes of the embryo.

## Materials and methods

### Animals, embryos and treatments

Adult sea urchins (*Paracentrotus lividus*) were collected in the bay of Villefranche-sur-Mer. Embryos were cultured as described in Lepage and Gache (1989, 1990) [64, 65]. Fertilization envelopes were removed by adding 1mM 3-amino-1,2,4 triazole (ATA) 1 min before insemination to prevent hardening of this envelope followed by filtration through a 75 μm nylon net. Treatments with U0126 (Calbiochem, 10 μM) BIRB796 (Selleckchem, S1574, 3 μM), SP600125 (Tocris, 0,5 μM), SB431542 (Tocris, 1614, 10 μM) and MG132 (Tocris 10 μM) were performed by adding the drug from stocks prepared in DMSO. Recombinant Nodal protein (R&D) was used at 1 μg/ml, NiCl_2_ was used at 0,2–0,3 mM and LiCl_2_ at 30mM-50mM.

In control treatments, embryos were treated with DMSO alone.

### Cloning of the Yan/Tel cDNA and sequences

A partial *yan/tel* cDNA was first amplified by PCR with degenerate primers corresponding to the ETS domain. A full-length *yan/tel* cDNA was subsequently obtained by screening a cDNA library with conventional methods and sequencing the corresponding clones. The 5' end of the cDNA was obtained by performing 5’RACE using the Smart RACE kit (Clontech). The accessions numbers of the Yan/Tel, β-TRCP and GSK3 mRNA are respectively: KF442410, MG719522, CAA10901.

### Oligonucleotides for cloning into pCS2, for mutagenesis of the phosphorylation sites and for insertion of the HA-epitope tags

Oligonucleotides for making the pCS2 *yan/tel* and pCS2 β-TRCP construct are:

Yan EcoRI-ATG fw: 5'-AGGGAATTCACCATGGATCCAGCATCGGCC-3'

Yan-TAG XbaI rev: I 5'-TGCTCTAGACTAGGTCTCCATCTCGGGTGA-3'

β-TRCP ClaI ATG fw: 5'- GGGATCGATACCATGGAGACCAGTACTATCAATGAAG-3'

β-TRCP TAG XhoI rev 5'- AGGCTCGAGCTATCGACAACTATTAGATACATATG-3'

All PCR reactions were made using high fidelity DNA polymerases and the constructs were entirely verified by sequencing.

To make the *yan/tel* rescue construct, an oligonucleotide containing 5 mismatches in the sequence recognized by the morpholino was used to amplify the coding sequence. The sequence of this oligonucleotide is:

5'-CGCGAATTCACCATGGACCCGGCGAGTGCTAGGCAGGTTCATCACTC (mismatches underlined)

The following oligonucleotides were used to mutate the putative phosphorylation sites of Yan/Tel (the mutated codon is in bold)

Yan MAPK1 S155A fw

5'-CAGCATGTTCCTGGA**GCA**CCACGGGAACCGATTG-3'

Yan MAPK2 S273A fw

5'-ACCCCAGTGCCGCCG**GCG**CCGACGAAGCGTATC-3'

Yan MAPK3 S733A fw

5'- CCCAATAGTAGACCACAG**GCA**CCCGAGATGGACACCTAG

Yan β-TRCP mut fw

5'- GAC**GCT**GGCCACAGT**GCC**CCCGACACGGCAGAAGAC

Yan cluster mut fw

5'- GTTCACATGGCTTCG**GCG**CCAACA**GCG**CCC**GCC**CCA**GCC**CCTCCCGTAGCT

Yan S12A fw

5'- GCTAGGCAGGTTCATCAC**GCA**CCGCATCCAATGATGCAG-3'

Yan S238A fw

5'- CACATCCTCCACTCGTAAC**GCA**CCCCAGCA-3'

Yan S594A

5'- CACATCCATCTCCACCTCTGCCCCATTGACTGTACCTTC-3'

Yan S632A

5'- GGCAGAGTCTACGCTCTATCA**GCT**CCATCCACAACTG

To introduce three copies of the HA epitope tag into Yan, the following oligos were used to amplify the whole pCS2-yan plasmid and the resulting PCR product was phosphorylated and religated. The resulting protein sequence is (HA tags are underlined and bold sequence from Yan/Tel N-terminus): MYPYDVPDYAGYPYDVPDYAGYPYDVPDYA**MDPASARQ**

3HA-Yan fw

-5’ TAC CCA TAC GAT GTT CCA GAT TAC GCT GGC TAT CCC TAT GAC GTC CCG GAC TAT GCA GGA TAT CCA TAT GAC GTT CCA GAT TAC GCT ATG GAC CCG GCG AGT GCT AGG CAG G-3’

Yan-reverse

-5' CATGGTGAATTCGAATCGATGGGATCCTGCAAAAAG-3'

### RNA extraction and northern blotting

Total RNA from staged embryos was extracted by the method of Chomczynski and Sacchi (1987) [66]. Samples of total RNA (20μg per lane) were fractioned on 1% agarose gel containing 0,66 M formaldehyde and transferred to membrane by standard methods (Sambrook et al., 1989) [67]. A P^32^ labeled RNA corresponding to the Yan/Tel ORF was used as a probe.

### In vitro phosphorylation assays on Yan/Tel fragments

To perform the in vitro kinase assays, fragments of Yan were cloned into pGex4T and the resulting fusion proteins were purified on glutathione sepharose affinity columns. As a positive control, we used a peptide corresponding to the N-terminal region of human β-catenin which contains a β-TRCP phosphodegron followed by GSK3 and casein kinase phosphorylation sites.

To insert the Yan β-TRCP and ß-catenin N-terminal sequences into the pGex4T vector, the following oligonucleotides were used: After amplification, the PCR product was phosphorylated and religated using the Q5 PCR kit from Biolabs.

Yan β-TRCP wt-pGex4T1-fw (PCSDSGHSSPDTA)

5'-CCGTGCAGTGACTCTGGCCACAGTTCCCCCGACACGGCA**TTCCCGGGTCGACTCGAGCGG**-3'

Yan β-TRCP3SA-pGex4T1-FW (PCSDAGHAAPDTA)

CCGTGCAGTGACGCTGGCCACGCTGCCCCCGACACGGCA**TTCCCGGGTCGACTCGAGCGG**-3'

Yan cluster wt-pGEX4T1-fw (STTPSPSPPVA)

5'-TCAACAACGCCCTCCCCATCCCCTCCTGTAGCC**TTCCCGGGTCGACTCGAGCGG**-3'

Yan cluster 3A-pGEX4T1-fw (STAPAPAPPVA)

5'-TCAACAGCGCCCGCCCCAGCCCCTCCTGTAGCC**TTCCCGGGTCGACTCGAGCGG**-3'

β-catenin- pGEX4T1-fw (SYLDSGIH**S**GAT**T**APC)

5'TCCTACCTCGATTCCGGTATCCATTCCGGAGCAACAACAGCA**TTCCCGGGTCGACTCGAGCGG**-3'

For the in vitro kinase reactions, 15 micrograms of each GST-fused Yan/Tel fragment was incubated for 3 h at 37°C with 250 units of recombinant GSK3 (Biolabs P6040S) and 500 units of Casein kinase (Biolabs P6030S) in 25 μl of 50 mM Tris pH: 7.5, 10 mM MgCl_2_, 0.1 mM EDTA, 2 mM DTT, 0.01% Brij 35 supplemented with 1mM ATP. The reactions were terminated by addition of SDS sample buffer and an aliquote was subjected to SDS-PAGE.

### Western blotting

Protein samples equivalent to 600 embryos per well for controls and treated embryos or to 50 embryos for embryos injected with 400ng of HA-Yan/Tel mRNA were separated by SDS-gel electrophoresis and transferred to PVDF membranes. After blocking in 5% dry milk blots were incubated overnight with the primary antibody diluted in 5% BSA in TBST. After washing and incubation with the secondary antibody, bound antibodies were revealed by ECL immunodetection using the SuperSignal West Pico Chemiluminescent substrate (Pierce) and imaged with a Fusion Fx7.

Antibodies used:

- anti-GSK3ß (phospho Y216)(abcam, ref ab75745)
- anti-Phospho-p38MAPK(Thr180-Tyr182)(Cell signaling, D3F9, ref.4511)
- anti-Phospho-p44/42 MAPK(Erk1/2)(Thr202/Tyr204)(Cell signaling, D13.14.4E, ref.4370)
- anti-Phospho-SAPK/JNK(Thr183/Tyr185)(Cell signaling, ref. 9251)
- anti-HA (Roche, ref. 3F10)
- anti-ATF2-P (Biolabs, ref. 9221)
- anti-β-Actin (Cell signaling, ref. 4967)

### Immunostaining

Embryos were fixed with 4% formaldehyde for 15 min then briefly permeabilized with methanol. Anti-Phospho-p38-MAPK (Thr180-Tyr182) was used at 1/50. Anti-Phospho-p44/42 MAPK (Erk1/2)(Thr202/Tyr 204) was used at 1/300. Embryos were imaged with an Axio Imager.M2 microscope.

### In situ hybridization

Probes derived from pBluescript vectors were synthesized with T7 RNA polymerase after linearization of the plasmids by NotI, while probes derived from pSport were synthesized with SP6 polymerase after linearization with XmaI. The *nodal*, *lefty*, *chordin*, *bmp2/4*, *tbx2/3*, *gata1/2/3*, *gcm* and *29D* probes have been described previously [1, 2, 34, 35, 68].

In situ hybridization was performed using standard methods (Harland, 1991) with DIG-labeled RNA probes and developed with NBT/BCIP reagent. Detection of the lineage tracer was performed using an anti-fluorescein antibody coupled to alkaline phosphatase and using Fast Red as substrate. Control and experimental embryos were developed for the same time in the same experiments. Embryos were imaged with an Axio Imager.M2 microscope.

### Overexpression of mRNAs and morpholino injections

For overexpression studies, capped mRNAs were synthesized from NotI-linearized templates using mMessage mMachine kit (Ambion). After synthesis, capped RNAs were purified on Sephadex G50 columns and quantitated by spectrophotometry. RNAs were mixed with Tetramethylrhodamine Dextran (10000 MW), Texas Red Dextran (70000 MW) or Fluoresceinated Dextran (70000 MW) at 5 mg/ml and injected in the concentration range 100–1000μg/ml. Wild-type *yan/tel* and mutated *yan/tel* mRNAs were injected at 900–1000 μg/ml. β*trcp* mRNA at 1000 μg/ml, *gsk3*β mRNA at 400 μg/ml, *panda* mRNA at 1000 μg/ml, *nodal* mRNA at 400 μg/ml and *alk457Q265D* mRNA at 400 μg/ml

Morpholino oligonucleotides were dissolved in sterile water and injected at the one-cell stage together with Tetramethylrhodamine Dextran (10000 MW) or Fluoresceinated Dextran (FLDX) (70000 MW) at 5 mg/ml. For each morpholino a dose-response curve was obtained and a concentration at which the oligomer did not elicit non-specific defects was chosen. Approximately 2–4 pl of oligonucleotide solution were injected in the experiments described here. Sequences for morpholino oligonucleotides used in this study are:

*yan/tel*-Mo5’ UTR: 5'-ATGGTGTCAGGAGTGGGATCAACAC-3'

*yan/tel*-Mo splice: 5'-GGGACTACATACCTATACGTGAGCT-3'

*yan/tel*-Mo 5' ATG: 5'-GATGCTGGATCCATAGGGCTTTGAA-3'

*lefty*-Mo: 5'-GGAGCGCCATGAGATAATTCCATAT-3'

*foxQ2*-Mo: 5'-GTTATCAATGCTGAACAGAGTCATG-3'

*panda* Mo ATG: 5'-ATCTTTGGAATGTGCGTCGAGCCAT -3'

The *yan/tel* morpholino was used at 0.75 mM, the *lefty* morpholino at 1.5 mM, the *foxQ2* morpholino at 1mM and the *panda* morpholino at 1mM. In the experiment using suboptimal doses of the Yan and panda morpholinos, these reagents were used respectively at 0.5 and 0.6 mM.

All the injections were repeated multiple times and for each experiment >50 embryos were analyzed (see supporting S1 Table for more detail of the number of experiments and number of embryos analyzed). Only representative phenotypes present in at least 80% of the injected embryos are presented.

### Mutagenesis of the *nodal* promoter

The conserved predicted binding sites for ETS factors in the R2 module of the *nodal* promoter [4] were identified using the TransFac software and the MatInspector software from Genomatix. Mutations were introduced by PCR using the Quick Change site directed Mutagenesis kit from Stratagene. All mutations were confirmed by restriction digestion and sequencing.

The following primers were used for mutagenesis of ETS sites (bold letters indicate the mutations introduced, restrictions sites are underlined):

Ets mut 1,2 Fw XhoI:

5’- CACATCTCTTCGTTTTT**AA**GAAAA**C**TC**GA**GATGATTAATTAGTATG-3’

Ets mut 1,2 Rev XhoI:

5’- CATACTAATTAATCATC**TC**GA**G**TTTTC**TT**AAAAACGAAGAGATGTG-3’

Ets mut 3 Fw XbaI:

5’- TTAATATTCATTAAATCT**AGA**GTCACTCGTTTTCTTACTT-3’

Ets mut 3 Rev XbaI:

5’- AAGTAAGAAAACGAGTGAC**TCT**AGATTTAATGAATATTAA-3’

Ets mut 4 Fw:

5’-GATTCATTGTTAATTAG**TCA**GACGGGTTGGGGAGATGGGTTCCTTGTG-3’

Ets mut 4 Rev:

5’-CACAAGGAACCCATCTCCCCAACCCGTC**TGA**CTAATTAACAATGAATC-3’

Ets-mut 5 Fw MluI:

5’-GGTTGATCAATAC**ACG**C**G**TTGTGTAGTGGGCCGA-3’

Ets mut 5 Rev Rev MluI

5’- TCGGCCCACTACACAA**C**G**CGT**GTATTGATCAACC-3’

## Supporting information

S1 Fig Structure of the sea urchin *yan/tel* mRNA and Yan/Tel protein. A, Structure of the *yan/tel* transcript and deduced Yan/Tel protein sequence. The predicted protein contains a SAM domain, an ETS binding site domain and several MAPK consensus phosphorylation sites indicated as S or T. The three canonical consensus MAPK phosphorylation sites are highlighted in yellow and the cluster of 4 phosphorylation sites is highlighted in blue. B, The hydrophobic residues involved in polymerization between monomers of Yan from *Drosophila* or Tel from vertebrates are conserved in the sea urchin Yan/Tel protein (highlighted in green and grey). C, Sequence alignments between Yan/Tel proteins from sea urchin (Pl, *Paracentrotus lividus*, Sp, *Strongylocentrotus purpuratus*), human (Hs, *Homo sapiens*), mouse (Mm, *Mus musculus*), zebrafish (Dr, *Danio rerio*) and flies (Dm, *Drosophila melanogaster* and Dv, *Drosophila viridis*). The positions of the SAM domain, the β-TRCP consensus and ETS domain are highlighted in green, blue and red, respectively. Conserved phosphorylation sites are identified by the position of red arrows.
(TIF)

S2 Fig Temporal expression of *yan/tel* during development. A, Northern blot of total RNA prepared at the indicated stages. (egg), unfertilized egg; (16), 16-cell stage; (64), 64-cell stage; (EB), early blastula; (MB), mesenchyme blastula; (EG), early gastrula; (LG), late gastrula; (Pr), prism; (Pl), pluteus. The blot was probed with a DNA fragment corresponding to the whole cDNA sequence (including the UTRs). B, Ethidium bromide staining of the corresponding gel.
(TIF)

S3 Fig Specificity of the *yan/tel* morpholino. A, Rescue experiment to control for the specificity of the translation blocking *yan/tel* morpholino. While embryos injected with the *yan/tel* morpholino are radialized and lack a skeleton, embryos co-injected with the *yan/tel* morpholino and a synthetic *yan/tel* mRNA immune against the morpholino develop with a normal dorsal-ventral axis and contain spicules. (hpf), hours post-fertilization. B, While all the embryos injected with the *yan/tel* morpholino display massive ectopic expression of *nodal*, in most (>90%) embryos co-injected with the *yan/tel* morpholino and the synthetic *yan/tel* mRNA, *nodal* expression is restricted to a discrete sector of the ectoderm. vv, vegetal view. lv, lateral view. In lateral views, animal is to the top, and ventral to the left.
(TIF)

S4 Fig Inhibition of zygotic Yan/Tel function does not perturb dorsal-ventral axis formation. In contrast to inhibition of maternal *yan/tel* function (see Fig 2), inhibition of zygotic *yan/tel* function does not perturb dorsal-ventral axis formation and *nodal* expression. Injection of the Yan/Tel splice morpholino however disrupts skeletogenesis consistent with the expression of Yan/Tel in the skeletogenic mesenchyme lineage. SB, swimming blastula stage; vv, vegetal view.
(TIF)

S5 Fig Western blot of JNK, ATF2, ERK, and p38 activation after treatment with increasing concentrations of the JNK inhibitor SP600125. Western blot analysis at hatching blastula stage of control and embryos treated with increasing concentrations of the SP600125 inhibitor during 30 minutes. Note that although the activation of JNK (P-JNK) is not perturbed by treatment with the inhibitor, the activity of JNK measured by its ability to phosphorylate ATF2 after an osmotic shock is suppressed in the presence of the inhibitor starting at 1μM.
(TIF)

S1 Table Biological replicates, technical replicates and number of embryos analyzed in all the experiments presented in this paper. (DOCX)
