# Supplementary figures and images for: MAPK and GSK3/ß-TRCP-mediated degradation of the maternal Ets domain transcriptional repressor Yan/Tel controls the spatial expression of nodal in the sea urchin embryo
Source: PLoS Genet. 2018 Sep 17;14(9):e1007621. doi: 10.1371/journal.pgen.1007621 (PMC6160229; doi:10.1371/journal.pgen.1007621)

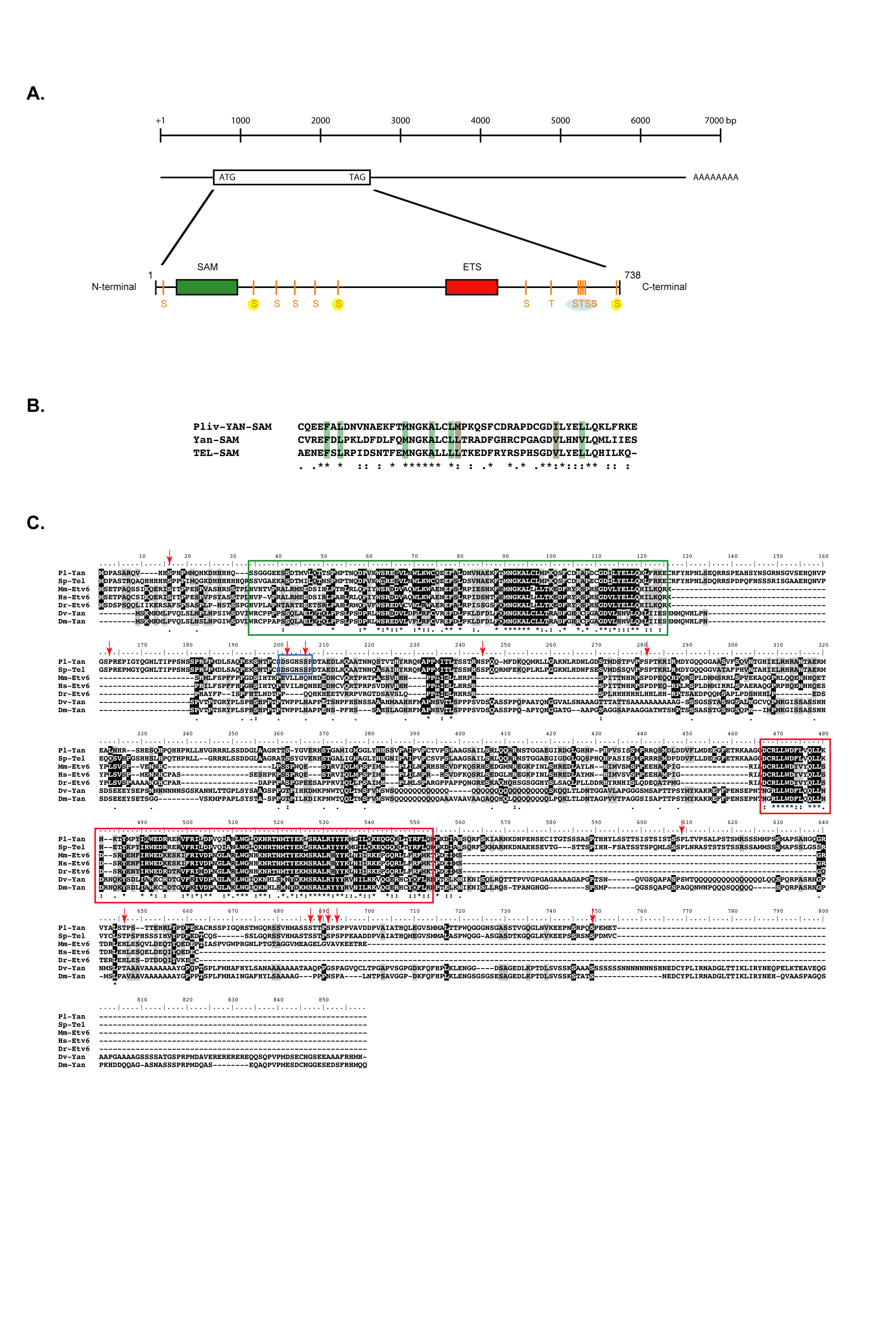

Supplement: S1 Fig — A, Structure of the yan/tel transcript and deduced Yan/Tel protein sequence. The predicted protein contains a SAM domain, an ETS binding site domain and several MAPK consensus phosphorylation sites indicated as S or T. The three canonical consensus MAPK phosphorylation sites are highlighted in yellow and the cluster of 4 phosphorylation sites is highlighted in blue. B, The hydrophobic residues involved in polymerization between monomers of Yan from Drosophila or Tel from vertebrates are conserved in the sea urchin Yan/Tel protein (highlighted in green and grey). C, Sequence alignments between Yan/Tel proteins from sea urchin (Pl, Paracentrotus lividus, Sp, Strongylocentrotus purpuratus), human (Hs, Homo sapiens), mouse (Mm, Mus musculus), zebrafish (Dr, Danio rerio) and flies (Dm, Drosophila melanogaster and Dv, Drosophila viridis). The positions of the SAM domain, the β-TRCP consensus and ETS domain are highlighted in green, blue and red, respectively. Conserved phosphorylation sites are identified by the position of red arrows. (TIF) [file pgen.1007621.s001.tif]

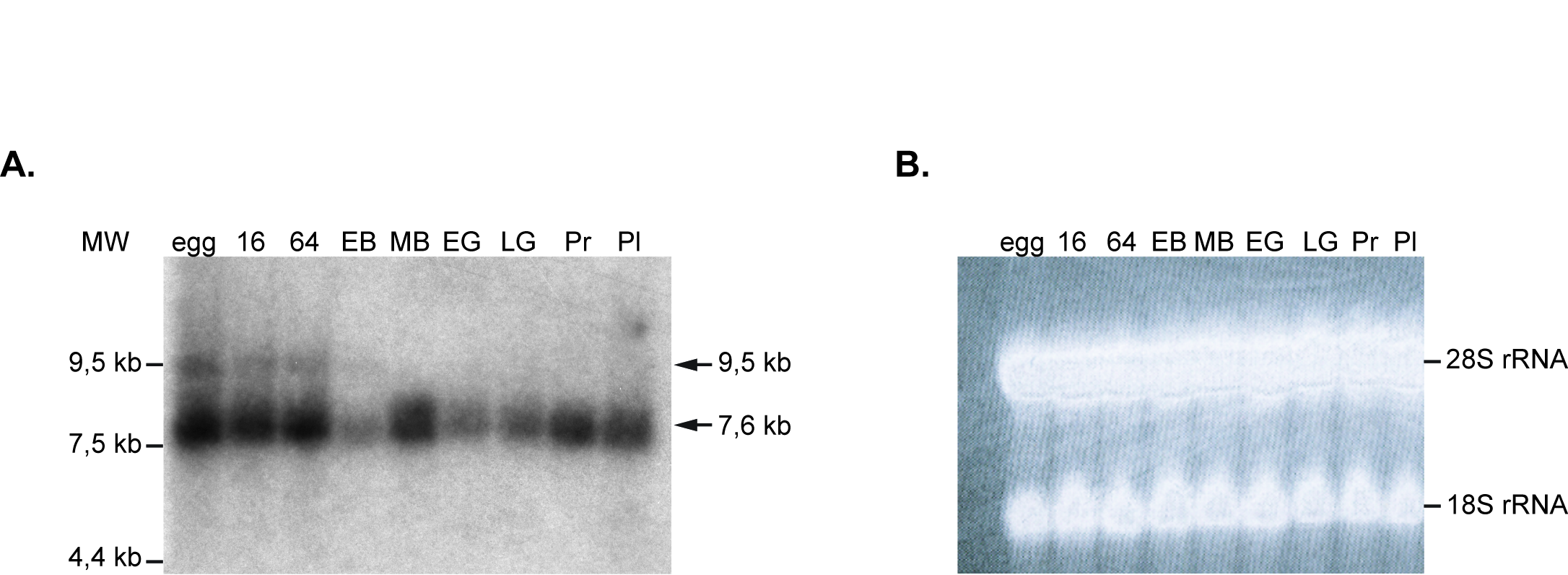

Supplement: S2 Fig — A, Northern blot of total RNA prepared at the indicated stages. (egg), unfertilized egg; (16), 16-cell stage; (64), 64-cell stage; (EB), early blastula; (MB), mesenchyme blastula; (EG), early gastrula; (LG), late gastrula; (Pr), prism; (Pl), pluteus. The blot was probed with a DNA fragment corresponding to the whole cDNA sequence (including the UTRs). B, Ethidium bromide staining of the corresponding gel. (TIF) [file pgen.1007621.s002.tif]

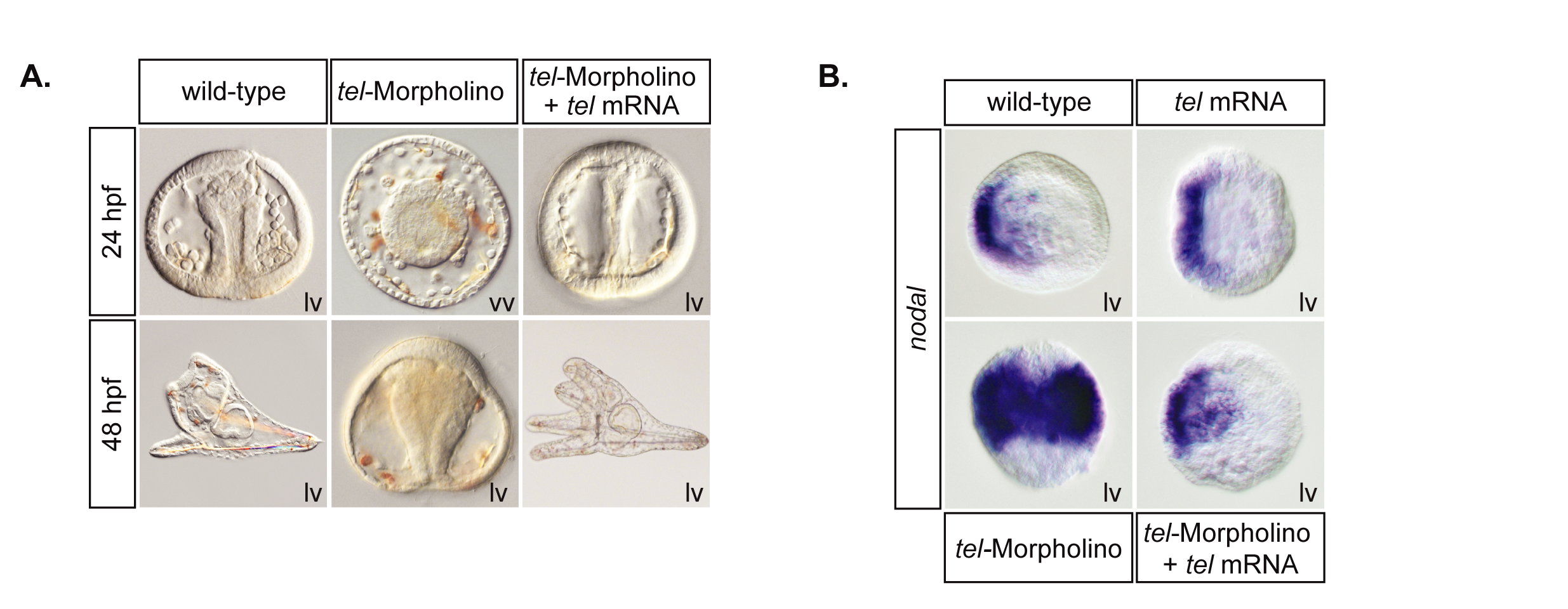

Supplement: S3 Fig — A, Rescue experiment to control for the specificity of the translation blocking yan/tel morpholino. While embryos injected with the yan/tel morpholino are radialized and lack a skeleton, embryos co-injected with the yan/tel morpholino and a synthetic yan/tel mRNA immune against the morpholino develop with a normal dorsal-ventral axis and contain spicules. (hpf), hours post-fertilization. B, While all the embryos injected with the yan/tel morpholino display massive ectopic expression of nodal, in most (>90%) embryos co-injected with the yan/tel morpholino and the synthetic yan/tel mRNA, nodal expression is restricted to a discrete sector of the ectoderm. vv, vegetal view. lv, lateral view. In lateral views, animal is to the top, and ventral to the left. (TIF) [file pgen.1007621.s003.tif]

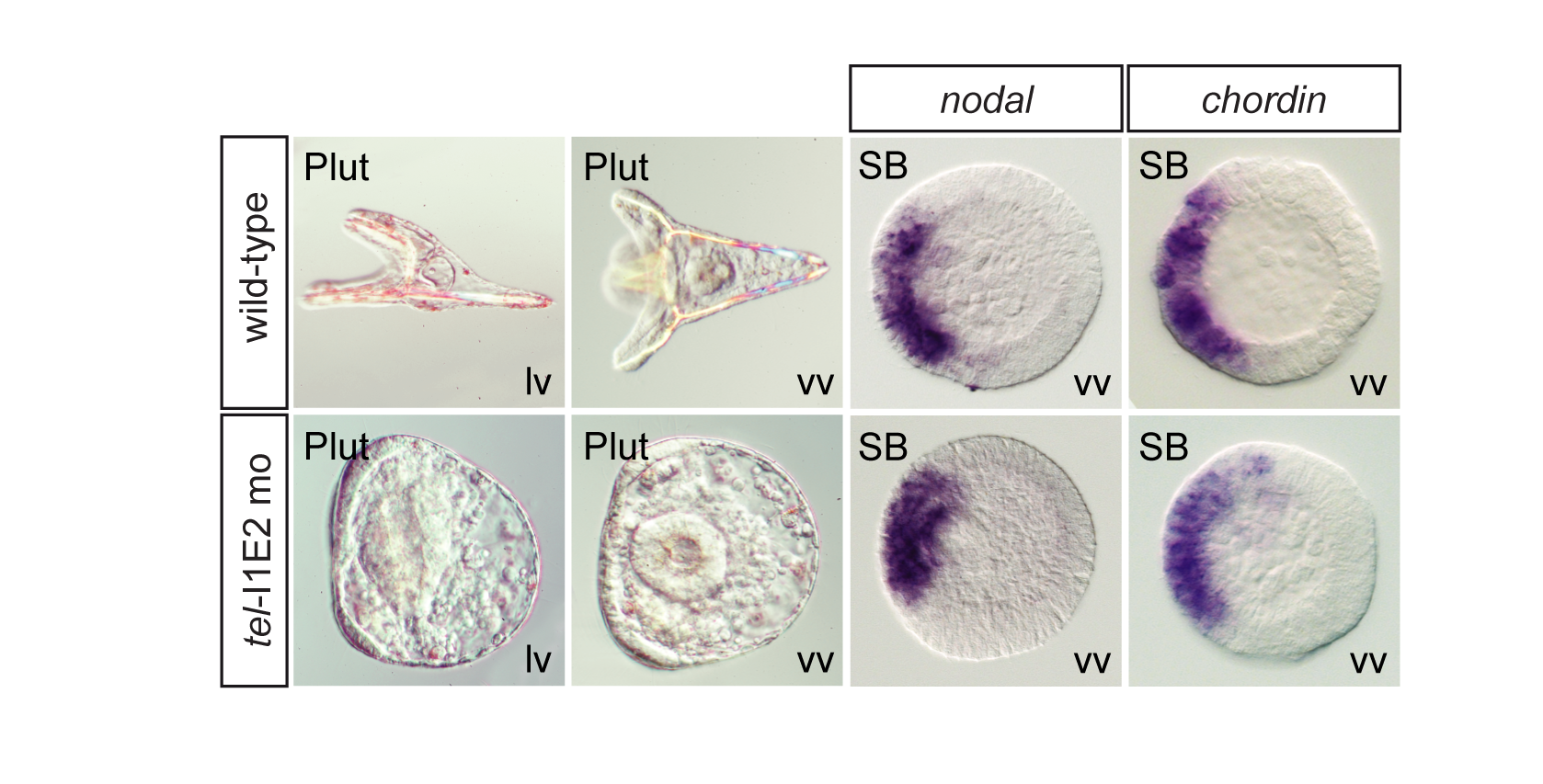

Supplement: S4 Fig — In contrast to inhibition of maternal yan/tel function (see Fig 2), inhibition of zygotic yan/tel function does not perturb dorsal-ventral axis formation and nodal expression. Injection of the Yan/Tel splice morpholino however disrupts skeletogenesis consistent with the expression of Yan/Tel in the skeletogenic mesenchyme lineage. SB, swimming blastula stage; vv, vegetal view. (TIF) [file pgen.1007621.s004.tif]

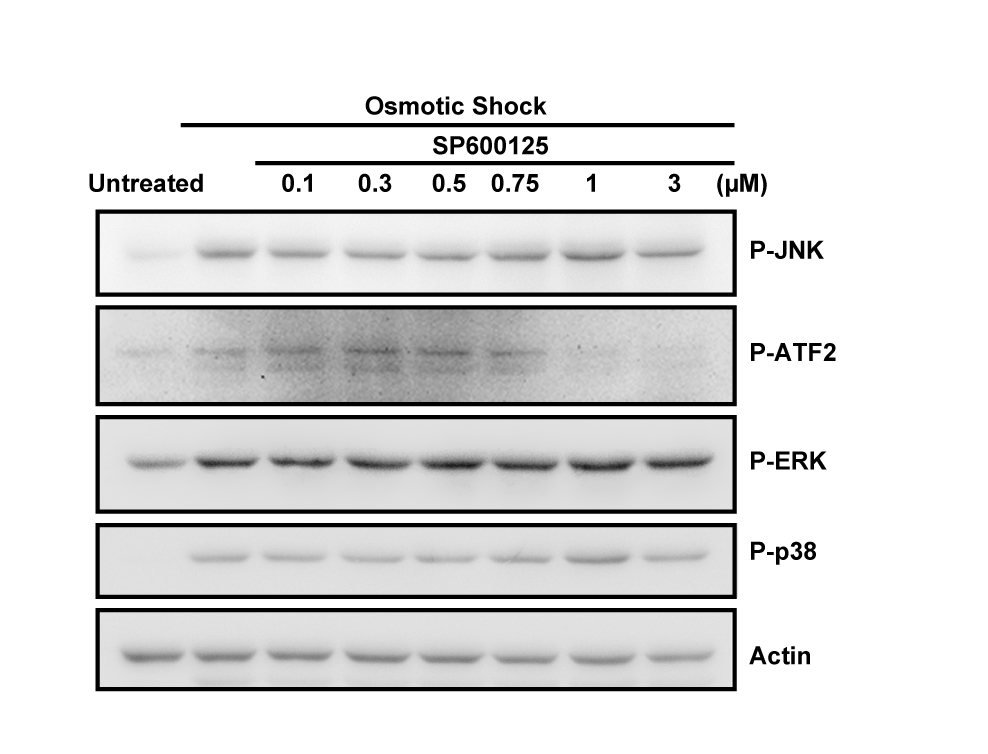

Supplement: S5 Fig — Western blot analysis at hatching blastula stage of control and embryos treated with increasing concentrations of the SP600125 inhibitor during 30 minutes. Note that although the activation of JNK (P-JNK) is not perturbed by treatment with the inhibitor, the activity of JNK measured by its ability to phosphorylate ATF2 after an osmotic shock is suppressed in the presence of the inhibitor starting at 1μM. (TIF) [file pgen.1007621.s005.tif]
