# Supplementary material for: MAPK and GSK3/ß-TRCP-mediated degradation of the maternal Ets domain transcriptional repressor Yan/Tel controls the spatial expression of nodal in the sea urchin embryo
Source: PLoS Genet. 2018 Sep 17;14(9):e1007621. doi: 10.1371/journal.pgen.1007621 (PMC6160229; doi:10.1371/journal.pgen.1007621)
Supplement: S1 Table — (DOCX) [file pgen.1007621.s006.docx]

|  | Experiment | Number of experiments | Number of embryos | Comments |
| --- | --- | --- | --- | --- |
| Figure 2A | Tel mo | > 10 | > 500 | Several concentrations tested |
| Figure 2BC | In situ | > 10 | > 500 | For each probe |
| Figure 2D | In situ | 2 | 25-50 | For each probe |
| Figure 2E | In situ | 2 | 25-50 | For each probe |
| Figure 2F | Orient. assay | >6 | >100 |  |
| Figure 2F | In situ | 1 | 25-50 |  |
| Figure 2G | In situ | 4 | >100 | Several concentrations tested |
| Figure 3A | Lefty mo | 2 | 25-50 | For each time point |
| Figure 3A | Tel mo | 1 | 25-50 | For each time point |
| Figure 3B | FoxQ2 mo | >5 | 100-150 |  |
| Figure 3B | Tel mo | >10 | >300 |  |
| Figure 3B | Panda mo | >5 | >200 |  |
| Figure 3B | Lefty mo | >5 | >150 |  |
| Figure 3B | FoxQ2 mo  + Tel mo | 3 | 50-100 |  |
| Figure 3B | FoxQ2 mo  + Panda mo | 4 | 75-100 |  |
| Figure 3B | Lefty mo  + Tel mo | 2 | 50-75 |  |
| Figure 3B | Lefty mo  + Panda mo | 4 | 75-100 |  |
| Figure 4B | Tel mRNA | >10 | >150 |  |
| Figure 4B | Tel Btrcp^mut^ mRNA | >6 | >100 |  |
| Figure 4B | Tel MAPK^mut^ mRNA | >6 | >100 |  |
| Figure 4B | Tel cluster^mut^ mRNA | >6 | >100 |  |
| Figure 4B | Tel 8A  mRNA | >10 | >150 |  |
| Figure 4B | Tel 10A mRNA | >6 | >100 |  |
| Figure 4B | Tel 13A mRNA | >10 | >150 |  |
| Figure 4C | In situ | 2 | 25- 175 | Check Figure 4C for details |
| Figure 4D | Western Blot 3HATel wt | >10 | 50 | For each experiment |
| Figure 4D | Western Blot  3HATel beta-  trcp | 2 | 50 | For each experiment |
| Figure 4D | Western Blot 3HATel MAPK | 2 | 50 | For each experiment |
| Figure 4D | Western Blot 3HATel Cluster | 2 | 50 | For each experiment |
| Figure 4D | Western Blot 3HATel 13A | >10 | 50 | For each experiment |
| Figure 4E | Orient. assay | >5 | >100 |  |
| Figure 4E | In situ | 2 | 20-40 |  |
| Figure 5A | Western Blot | 3 | 50 | For each experiment |
| Figure 5B | In situ | 3 | >50 | For each condition/experiment |
| Figure 5C | Western Blot | 2 | 500 | For each condition/experiment |
| Figure 5D | IF P-ERK | 3 | >50 |  |
| Figure 5D | IF P-p38 | >5 | >50 |  |
| Figure 5E | In situ | 1 | 25-50 | For each condition/experiment |
| Figure 5F | Western Blot | 2 | 50 | For each condition/experiment |
| Figure 5G | Western Blot | 3 | 50 | For each condition/experiment |
| Figure 6C | Western Blot  Tel mRNA  +LiCl | >5 | 50 | For each experiment |
| Figure 6C | Western Blot Tel 10A mRNA  +LiCl | 2 | 50 | For each experiment |
| Figure 6C | Western Blot  Tel mRNA  + GSK3 mRNA | 3 | 50 | For each experiment |
| Figure 6C | Western Blot  Tel 10A mRNA  + GSK3 mRNA | 1 | 50 |  |
| Figure 6D | Western Blot | 2 | 50 | For each experiment |
| Figure 6E | Western Blot | 2 | 500 | For each experiment |
| Figure 6F | In situ | 2 | >50 | For each probe |
| Figure 6G | In situ | 2 | 25-50 | For each condition |
| Figure 6H | Western Blot | 2 | - |  |
| Figure 7A | Orient. assay | 2 | 10-25 |  |
| Figure 7B | In situ | 1 | 10-60 |  |
| Figure 7C | Western Blot | 3 | 50 | For each condition/experiment |
| Figure 7D | Tel mo  + Panda mo | 4 | 30-60 | For each experiment |
| Figure 7D | In situ | 2 | 25-50 | For each experiment |
| SuplFig 2 | Northern blot | 2 |  |  |
| SuplFig 3A | Tel mo rescue | 2 | 25-50 |  |
| SuplFig 3B | In situ | 2 | 25-50 |  |
| SuplFig 4 | Tel mo intron | >3 | 25-30 | For each experiment |
| SuplFig 4 | In situ | 3 | 25-50 | For each experiment and probe |
| SuplFig 5 | Western blot | 3 | 500 | For each condition |
